# Engineered extracellular vesicles for tissue repair and regeneration

**DOI:** 10.1093/burnst/tkae062

**Published:** 2024-10-22

**Authors:** Yan Zhang, Dan Wu, Chen Zhou, Muran Bai, Yucheng Wan, Qing Zheng, Zhijin Fan, Xianwen Wang, Chun Yang

**Affiliations:** College of Basic Medicin, Beihua University, No. 3999 Binjiang East Road, Fengman District, Jilin City, Jilin Province, China; School of Public Health, Beihua University, No. 3999 Binjiang East Road, Fengman District, Jilin City, Jilin Province, China; College of Basic Medicin, Beihua University, No. 3999 Binjiang East Road, Fengman District, Jilin City, Jilin Province, China; Department of Laboratory Medicine, The Eighth Affiliated Hospital, Sun Yat-Sen University, No. 3025 Shennan Middle Road, Futian District, Shenzhen, China; College of Basic Medicin, Beihua University, No. 3999 Binjiang East Road, Fengman District, Jilin City, Jilin Province, China; Hospital of Stomatology, Zunyi Medical University, No. 89, Wujiang East Road, Xinpu New District, Zunyi City, Guizhou Province, China; College of Basic Medicin, Beihua University, No. 3999 Binjiang East Road, Fengman District, Jilin City, Jilin Province, China; Institute for Engineering Medicine, Kunming Medical University, No. 1168 Chunrong West Road, Yuhua Street, Chenggong District, Kunming City, Yunnan Province China; School of Biomedical Engineering, Research and Engineering Center of Biomedical Materials, Anhui Medical University, No.81 Meishan Road, Shushan District, Hefei 230032, China; College of Basic Medicin, Beihua University, No. 3999 Binjiang East Road, Fengman District, Jilin City, Jilin Province, China

**Keywords:** Extracellular vesicles, Tissue repair, Tissue regeneration, Wound healing, Regenerative medicine

## Abstract

Extracellular vesicles (EVs) are heterogeneous membrane-like vesicles secreted by living cells that are involved in many physiological and pathological processes and act as intermediaries of intercellular communication and molecular transfer. Recent studies have shown that EVs from specific sources regulate tissue repair and regeneration by delivering proteins, lipids, and nucleic acids to target cells as signaling molecules. Nanotechnology breakthroughs have facilitated the development and exploration of engineered EVs for tissue repair. Enhancements through gene editing, surface modification, and content modification have further improved their therapeutic efficacy. This review summarizes the potential of EVs in tissue repair and regeneration, their mechanisms of action, and their research progress in regenerative medicine. This review highlights their design logic through typical examples and explores the development prospects of EVs in tissue repair. The aim of this review is to provide new insights into the design of EVs for tissue repair and regeneration applications, thereby expanding their use in regenerative medicine.

## Graphical Abstract

**Graphical Abstract:**
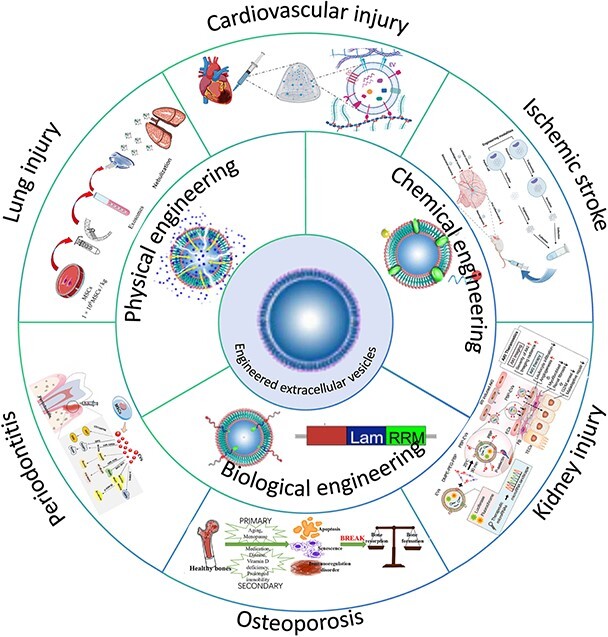

Highlights

- EVs have been described as a cell-free tissue repair strategy.
- Rational designs can improve therapeutic potential of EVs in tissue repair.
- Research advances in tissue repair with engineered EVs.
- Challenges and prospects of EVs in clinical transformation of tissue engineering.

## Background

Effective tissue repair and regeneration following injury are vital for maintaining the functionality and for the survival of all living organisms [1, 2]. However, unlike nonmammalian vertebrates, mammalian and human tissues and organs possess a limited capacity for self-regeneration, primarily due to differences in genetics, developmental processes, immune responses, and tissue complexity. A recent research has highlighted the potential of endogenous stem cells, which are tissue-specific adult stem cells capable of self-renew and differentiating into specific cell types [3]. While the use of endogenous stem cells offers distinct advantages over exogenous stem cells, such as a lower risk of immune rejection and oncogenesis, stem cell therapy still faces significant challenges and limitations [4]. Successfully achieving tissue regeneration after a disease or injury continues to be a major hurdle in the field of regenerative medicine.

In recent years, extracellular vesicles (EVs) have garnered considerable attention for their crucial role in intercellular communication and their promising potential in drug delivery research. These nanoscale extracellular structures, enclosed by lipid bilayers, are released by various cell types and are characterized by their size, density, biochemical composition, and their condition or origin. EVs are of interest not only because they facilitate intercellular communication but also due to their complex biochemical components and their significant impact on health and disease. Increasingly, researchers are exploring the capacity of EVs for tissue repair and regeneration, particularly those derived from stem cells. Stem cell-derived EVs hold substantial promise for tissue repair and regeneration, offering advantages such as the ability to evade immune responses in both *in vitro* and *in vivo* environments and the potential to differentiate into a wide range of specific cell types [5]. Mesenchymal stem cells (MSCs), which are pluripotent with multidifferentiation potential and self-renewal ability, have demonstrated potential in regenerating various tissues, organs, and cells [6, 7]. In addition to their use in restoring hematopoietic function and treating autoimmune diseases, MSCs are widely applied in the repair of various tissue damage, including skin, bone, cartilage, heart, and nerve tissues [8–12]. However, as stem cell research advances, it has become evident that stem cell therapy is a double-edged sword: while it can promote tissue repair, the uncertain multidirectional differentiation potential of stem cells poses a risk of tumorigenesis and raises significant ethical concerns [13, 14]. Increasing evidence suggests that the beneficial effects of MSCs may be largely attributed to their release of EVs and their paracrine effects rather than direct cell implantation and response to injury. These findings indicate that MSC-derived EVs (MSC-EVs) could offer the same therapeutic benefits as MSCs. Unlike primitive MSCs, MSC-EVs do not have the capacity for self-replication, thereby mitigating safety concerns associated with cell therapy, such as uncontrolled cell proliferation and the risk of contamination with tumorigenic cells [5].

While the role of EVs in tissue repair and regeneration has attracted considerable attention and been discussed in various reviews [15–17], a systematic summary of methods to enhance the effectiveness of EVs through rational engineering design has been lacking. In this work, we comprehensively explore the engineering design logic of EVs, evaluating the advantages and disadvantages of these designs based on their performance in specific cases [18–23]. This review aims to serve as a reference for future research in the field. One approach involves immobilizing EVs within biomaterials through cross-linking or EV-binding sites, allowing for targeted delivery to injured tissues to promote healing and restore function.

Natural extracellular matrix (ECM) biomaterials, such as collagen and hyaluronic acid, can bind EVs via integrin and CD44 receptors, respectively, while synthetic biomaterials offer controlled porosity and degradation rates to regulate EV release [19]. This experiment demonstrated two ways of engineering EVs (EEVs) and their therapeutic effects on ischemic stroke. The results suggest that EEVs are more effective than natural EVs in the treatment of ischemic stroke and inform future research in this area [24]. Another study highlighted the efficacy of P-selectin binding peptide-engineered EVs, which deliver therapeutic microRNA (miRNA) and imaging agents specifically to damaged kidneys by binding to P-selectin on injured endothelial cells. This system not only aids in monitoring the severity of acute kidney injury (AKI) but also promotes renal recovery, suggesting its potential for personalized treatment and improved long-term outcomes in AKI patients [18]. Further research demonstrated the use of exosomes in combating osteoporosis. Compared with placebo treatments, exosome therapy increased bone mass, improved bone microstructure, and enhanced bone strength, indicating a shift toward bone regeneration driven by stem cell-derived exosomes [21]. In the context of periodontitis, another study investigated the mechanisms by which MSC-EVs facilitate bone regeneration. The study identified several key signaling pathways involved in this process, including the RANKL-RANK, Wnt, AMPK, AKT and ERK, NF-κB p65, p38 MAPK, Smad5/Runx2, and miR-1246/Nfat5 axes, suggesting that MSC-EVs may offer a promising therapeutic approach for inflammatory bone loss [22]. Moreover, a clinical study involving seven coronavirus disease 2019 (COVID-19) pneumonia patients treated with aerosolized exosomes derived from bone marrow-derived mesenchymal stem cell (BMSC) showed promising results. These exosomes were collected and purified through multiple ultrafiltration steps and administered via inhalation. The treatment led to significant absorption of lung lesions and reduced hospital stays in patients with mild COVID-19 pneumonia. The findings confirm that aerosolized BMSC-derived exosomes are a safe, effective, and straightforward treatment option, particularly beneficial in the early stages of the disease [23]. In addition to discussing the potential and successes of EV-based therapies, this review also addresses the challenges and limitations associated with their clinical application, proposing future research directions to overcome these obstacles.

## Review

### The overview of EVs

#### Biogenesis and physiological functions of EVs

EVs are lipid bilayer-encased vesicles secreted by cells, categorized into three primary types based on their biogenesis: exosomes, microvesicles, and apoptotic bodies. Exosomes, ranging from 30 to 150 nm in size, originate from the invagination of the plasma membrane and were first identified in sheep reticulocytes in 1983, later named “exosomes” by Johnstone in 1987 [25–27]. The biogenesis of exosomes involves three stages: first, the plasma membrane invaginates to form early sorting endosomes (ESEs). These ESEs then mature into late-sorting endosomes, or multivesicular bodies (MVBs), containing intraluminal vesicles (ILVs). Finally, the MVBs either fuse with lysosomes for degradation or with the plasma membrane, leading to the release of ILVs into the extracellular environment as exosomes. Once released, these exosomes are rapidly cleared from the human body [28].

**Figure 1:**
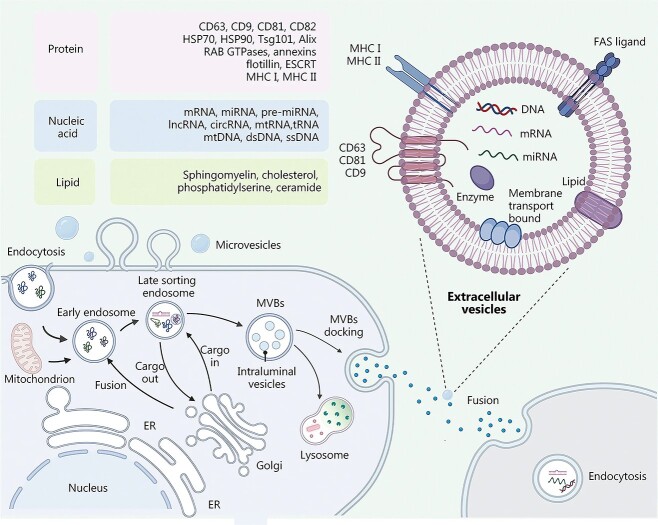
Composition, structure, and biogenesis of EVs. EVs are closed structures of phospholipid bilayers; various components of the extracellular environment, including proteins, nucleic acids, and lipids, are endocytosed to form early endosomes, which are later converted to late endosomes, which in turn form MVBs, which form exosomes by fusion with microtubules and the cytoskeleton with the plasma membrane; microvesicles are produced by cytosolic outgrowth; adapted with permission from ref [31]. Copyright 2023, the authors. *EVs* extracellular vesicles, *CD* cluster of differentiation, *HSP* heat shock protein, *Tsg* tumor susceptibility gene, *Alix* apoptosis-linked gene 2-interacting protein X, *RAB* Ras-like proteins in brain, *GTPases* guanosine triphosphate hydrolases, *ESCRT* endosomal sorting complex required for transport, *MHC* major histocompatibility complex, *mRNA* messenger ribonucleic acid, *miRNA* micro ribonucleic acid, *lncRNA* long noncoding ribonucleic acid, *mtRNA* mitochondrial ribonucleic acid, *tRNA* transfer ribonucleic acid, *dsRNA* double-stranded ribonucleic acid, *ssDNA* single-stranded deoxyribonucleic acid, *FAS* tumor necrosis factor receptor superfamily member 6, *DNA* deoxyribonucleic acid, *ER* endoplasmic reticulum

Microvesicles, which measure between 100 and 1000 nm, are primarily released by platelets and endothelial cells. They differ in size and shape and are formed by the outward budding of the plasma membrane. The largest EVs are apoptotic bodies, which range from 50 to 5000 nm in size and are released during programmed cell death. Traditionally associated with the clearance of cellular debris, apoptotic bodies are now recognized as key players in cell-to-cell communication, capable of transferring bioactive molecules and even entire organelles, such as mitochondria and ribosomes [29]. Among these types, exosomes and microvesicles have been the most extensively studied, leading to the term “EVs” often being used to refer specifically to these two subgroups [30]. Due to their rich content of proteins, nucleic acids, and lipids, EVs play critical roles in various biological processes, including intercellular communication, gene expression regulation, reproduction, cell development and proliferation, wound healing, metabolic regulation and reprogramming, signaling, immune response, apoptosis, and the progression of cancer [31] (Figure 1).

EVs have recently gained prominence as crucial intermediaries for intercellular information transfer and as vehicles for drug delivery across various biological systems. Pioneering research by Wood and colleagues demonstrated that EVs can selectively transport specific genetic cargo and target particular cell types through relatively straightforward methods [29, 32]. In addition, EVs play a vital role in fundamental biological processes by influencing pleiotropic functions. They achieve this through multiple mechanisms: directly activating cell surface receptors via proteins and bioactive lipid ligands, integrating their membrane contents into the plasma membrane of recipient cells, and delivering effectors such as transcription factors, oncogenes, small and large noncoding regulatory RNAs (including miRNAs), messenger RNAs (mRNAs), and even infectious particles. Through these diverse functions, EVs contribute to maintaining essential physiological processes, including stem cell maintenance, tissue repair, immune surveillance, and blood clotting. They can be viewed as multifunctional signaling complexes that regulate basic cellular and biological functions.

For instance, in immune response regulation, EVs can either initiate an adaptive immune response or suppress inflammation, depending on the condition of the specific immune cells involved [33–35]. EVs have been shown to confer immunosuppressive effects through several mechanisms: enhancing the function of regulatory T cells, inhibiting the activity of natural killer (NK) and CD8+ T cells, and preventing the differentiation and maturation of monocytes into dendritic cells. Conversely, EVs can also mediate immune activation, promote hematopoietic stem cell proliferation and survival, and stimulate monocytes, B cells, and NK cells. Notably, in 1996, it was first discovered that B-cell-derived EVs carry functional peptide-MHC complexes capable of directly presenting antigens to T cells. This discovery highlighted the role of EVs in antigen presentation, suggesting that they may play a significant role in adaptive immunity.

EVs have emerged as key players in tissue repair, demonstrating their ability to accelerate wound hemostasis, modulate macrophage polarization toward an anti-inflammatory state, stimulate the proliferation and migration of vascular endothelial cells and fibroblasts, regulate cytokine ratios, and remodel the ECM to facilitate tissue repair. Notably, EVs derived from MSCs have shown significant procoagulant effects on human blood and platelet-free plasma, underscoring their potential in wound healing [36]. Additionally, MSC-EVs have been implicated in reducing inflammation, particularly in hyperglycemic environments, by mitigating oxidative stress and inflammatory responses in diabetic mice [37]. This anti-inflammatory effect is thought to be mediated by the induction of M2 macrophage polarization and the reduction of pro-inflammatory cytokines such as TNF-α, IL-6, and IL-8 [38]. Beyond inflammation, EVs also enhance cell proliferation and angiogenesis. For instance, EVs derived from adipose-derived MSCs have been shown to increase the S-phase fraction of fibroblasts, thereby promoting their proliferation and contributing to skin regeneration. Furthermore, EVs play a role in ECM remodeling by inhibiting TGF-β1 and increasing the ratio of type III to type I collagen, TGF-β3 to TGF-β1, and MMP-3 to TIMP-1. This modulation of the ECM helps to prevent fibroblasts from differentiating into myofibroblasts via the TGF-β2/Smad2 pathway, thereby reducing scar formation and promoting wound healing [39].

The critical role of MSC-EVs in intercellular communication and their promising therapeutic potential have led to their development as alternative therapeutic options [40]. Currently, MSCs are widely used as regenerative agents in clinical research and the treatment of various conditions, including osteoarthritis, pulmonary fibrosis, spinal cord injury (SCI), myocardial injury, knee cartilage injury, pulp regeneration, and organ transplantation. The therapeutic efficacy of MSCs is largely attributed to their immunomodulatory functions, which are regulated by the inflammatory environment. Stimulated by inflammatory factors, MSCs produce a range of immunomodulatory molecules, cytokines, and growth factors that regulate the immune microenvironment and promote tissue regeneration. Emerging evidence suggests that MSC-EVs retain the therapeutic benefits of their parent MSCs while avoiding the safety concerns associated with live cell therapy, such as immune rejection and tumorigenesis [25]. EVs offer several advantages over cell-based therapies in tissue repair: they reduce immune risks associated with stem cell transplantation, have convenient storage conditions, can easily circulate through capillaries, and eliminate the risk of tumor formation [28]. In the context of neuroprotection, the beneficial effects of MSCs are partly due to the paracrine activity of their secreted factors, including inflammatory cytokines and neurotrophic factors. For example, Rajan *et al.* [41] demonstrated the neuroprotective effects of secreted factors from human gingival MSCs (hGMSCs) in a mouse model of motor neuron injury. They found that conditioned medium derived from hGMSCs significantly inhibited apoptosis, oxidative stress, and pro-inflammatory cytokines while upregulating neurotrophic factors and anti-inflammatory cytokines, suggesting its potential as an autotherapeutic tool for treating motor neuron injuries. Similarly, Cho et al. investigated the therapeutic potential of exosomes derived from human adipose tissue-derived mesenchymal stem cells (ASC-exosomes) in an *in vivo* mouse model of atopic dermatitis (AD). They found that ASC-exosomes, administered either intravenously or subcutaneously, significantly reduced pathological symptoms such as clinical scores, serum IgE levels, eosinophil counts, and inflammatory cell infiltration in skin lesions. ASC-exosomes also reduced the mRNA expression of various inflammatory cytokines in AD skin lesions, indicating their potential as a novel, cell-free therapy for AD treatment. In conclusion, the use of MSC-EVs as a free therapy may represent a future focus in clinical treatment, offering a safer and more efficient alternative to traditional MSC-based therapies [42].

#### Biochemical composition of EVs

EVs are natural carriers of biomolecules, including proteins, nucleic acids, and lipids, and they possess the unique ability to deliver these cargos to recipient cells both locally and over long distances. The bloodstream acts as a crucial transport medium, enabling EVs to travel throughout the body and efficiently transfer their contents to target cells while maintaining the functional integrity of the delivered molecules. This ability makes EVs highly promising candidates for drug delivery. In addition to their transport functions, EVs play a central role in intercellular communication, facilitating the exchange of materials and information between cells, thereby influencing a wide range of physiological processes [43].

##### Nucleic acid

miRNAs are potent regulators of gene expression, playing crucial roles not only within their original cellular environments but also in the regulation of gene expression across different biological systems. These small RNA molecules are detectable in various human body fluids, including plasma, serum, urine, saliva, and semen, with a portion encapsulated within the lumen of EVs. Since their discovery, miRNAs have been linked to key regenerative processes, such as cell proliferation, differentiation, migration, and apoptosis. Several miRNAs have emerged as promising candidates for inducing tissue regeneration and therapeutic effects across various organs. For instance, miR-124 and miR-9 have been shown to directly convert fibroblasts into neuron-like cells by downregulating BAF53a, a crucial component involved in neuronal development in mice during postmitotic growth [44]. Another miRNA, miR-126, has demonstrated regenerative potential by protecting cardiac muscle from apoptosis, oxidative stress, and fibrosis while also promoting cardiac repair.

Additionally, miR-92a-3p, present in exosomes derived from MSCs, has been found to enhance chondrogenesis and inhibit cartilage degeneration through the Wnt5A pathway, making it a promising therapeutic agent for osteoarthritis [45]. Exosomes from adipose-derived stem cells (ADSCs) carrying miR-375 have also shown potential by targeting the 3′ untranslated region of insulin-like growth factor binding protein 3 in bone marrow stem cells, promoting bone formation *in vitro*—a promising strategy for treating cranial defects [46]. Moreover, intravenous administration of MSC-EVs has been shown to prevent and reverse right ventricular hypertrophy and pulmonary vascular remodeling caused by acid–base disturbances. This is achieved through the delivery of specific miRNAs, such as miR-34a, miR-122, miR-124, and miR-127, which regulate anti-inflammatory and antiproliferative pathways [47].

While EVs have the capacity to enrich specific therapeutic miRNAs and mitigate pathological cellular states in various diseases, the development of precise methods for their clinical application remains a challenge. The drug delivery community and EV researchers continue to explore efficient techniques for large-scale EV isolation, miRNA loading, and safe delivery to target tissues. Further research into the endogenous mechanisms of miRNA sorting within EVs may unlock new therapeutic possibilities, paving the way for innovative treatments.

mRNA is a key component of EVs involved in tissue regeneration. Recent findings suggest that the regenerative effects of MSCs are largely attributed to their paracrine signaling mechanisms, specifically through the transfer of mRNA from donor cells to recipient cells via EVs [48]. This horizontal transfer of mRNA has been shown to influence immune regulation and tissue repair processes in recipient cells [49]. For example, in a murine model of hind limb ischemia, EVs containing mRNA for neural regulatory protein 1 from ADSCs were found to reduce muscle damage and inflammation [50]. Similarly, MSC-EVs carrying mRNA for vascular endothelial growth factor (VEGF-A), basic fibroblast growth factor (bFGF), and insulin-like growth factor 1 (IGF-1) have been reported to stimulate the proliferation of peritubular capillary endothelial cells in mice with acute renal ischemia. Furthermore, *in vitro* studies have demonstrated that the transfer of TGF-β mRNA via EVs can promote fibroblast proliferation and activation in tubular epithelial cells exposed to hypoxic injury, highlighting the role of EVs in modulating the microenvironment and enhancing tissue repair [51]. These examples underscore the potential of mRNA carried by EVs in promoting tissue repair and regeneration. However, more in-depth research is needed to fully understand the mechanisms and optimize the use of mRNA-loaded EVs for therapeutic applications in tissue repair and regeneration.

##### Proteins

Multiple studies have highlighted that the tissue regenerative effects of EVs are not solely attributable to miRNA and mRNA content but also to the diverse proteins they carry. These proteins play crucial roles in regulating both intracellular and extracellular environments of recipient cells. Proteomic analyses of MSC-EVs have revealed a range of proteins involved in key processes such as angiogenesis, coagulation, apoptosis, inflammation, ECM remodeling, and tissue repair [52, 53].

For example, MSC-EVs have been shown to enhance chondrocyte proliferation, migration, and ECM secretion through the expression of CD73, contributing to joint regeneration. Additionally, EVs derived from TGF-β-stimulated fibroblasts express PD-L1, which facilitates fibroblast migration through the action of EV-associated fibulin (FN). Furthermore, kidney cysts treated with hepatocyte growth factor (HGF) release EVs containing G protein-coupled receptor 5B (GPRC5B), which, in conjunction with HGF, promotes renal tubulogenesis [16].

EVs impact target cell behavior by modulating various signaling pathways, yet the intricate mechanisms underlying EV-mediated tissue remodeling and repair remain incompletely understood. To advance the development of EVs as cell-free therapeutic agents for organ repair, future research should address key questions regarding the specific roles of proteins in EV function and whether these roles are unique to certain EV subtypes. Continued investigation is essential to fully elucidate these mechanisms and enhance the therapeutic potential of EVs.

##### Lipids

EV lipids are critical components whose composition varies depending on the cell of origin. Key lipid constituents include cholesterol, sphingomyelin, ceramide, phosphatidylserine, and saturated fatty acids. Research by Skotland *et al.* [54] identified 107 lipids enriched in urine-derived EVs from prostate cancer patients, with 36 being notably abundant. Among these, nine specific lipids, including phosphatidylserine (PS) and lactose ceramide, were highly effective in distinguishing between patients and controls with exceptional sensitivity and specificity. This finding suggests their potential as valuable biomarkers for prostate cancer. Despite these insights, the role of lipid composition in EVs within the context of tissue regeneration remains underexplored. Understanding the detailed lipid profiles of EVs is crucial for comprehending their biological functions and optimizing their use in clinical applications. Future research should focus on elucidating how specific lipids contribute to the regenerative capabilities of EVs, which will aid in their development as therapeutic tools.

#### Isolation and characterization of EVs

Currently, methods for characterizing EVs include western blot (WB), microscopic analysis of morphology, and nanoparticle tracking analysis (NTA). Microscopy provides detailed images of EV structure, NTA allows for real-time monitoring of particle size, concentration, and movement, and WB is used to identify the expression of specific target proteins. EVs are categorized into subtypes based on their biogenesis pathway: exosomes, microvesicles, and apoptotic bodies. Each subtype differs in size, surface proteome, and cargo composition. For instance, exosomes are marked by proteins such as CD9, CD63, and CD81, which are involved in membrane fusion and signaling. Microvesicles feature membrane proteins like CD40, integrins, glycoprotein Ib, and P-selectin [55, 56]. Apoptotic bodies, which result from cellular apoptosis, contain a more complex mix of cytoplasmic and nucleoproteins, including calreticulin and calcineurin that aid in phagocytosis and phagolysosome formation, as well as receptors for platelet-activating protein and complement protein C3b, which enhance the recognition and clearance by phagocytes. These proteins serve as biomarkers for distinguishing between exosomes, microvesicles, and apoptotic bodies, aiding in clinical diagnosis and EV characterization [57]. However, separating EVs is challenging due to the overlap in size and density with other plasma components, such as lipoproteins (HDL, LDL, IDL, VLDL) and viruses. Improved techniques are needed to refine the separation and characterization of EVs from these similar entities [58].

Ultracentrifugation (UC) is currently the most widely used technique for exosome extraction and separation and is often referred to as the gold standard. UC separates components based on differences in particle size and density and is particularly effective for high-dose samples with significant variations in sedimentation coefficients. Despite its high yield and ease of use, UC faces limitations in clinical translation due to its low specificity, time-intensive nature, high cost, and potential to cause structural damage to exosomes [28].

Density gradient centrifugation (DGC) is frequently combined with UC to isolate EVs from biological fluids. DGC creates a density gradient using solutions like sucrose or iodixanol (OptiPrep), which allows for more refined separation of EVs [59]. Although DGC can produce purer EV samples compared with UC, it is hampered by the need for specialized equipment, high costs, and the presence of contaminants such as higher order protein aggregates and lipoproteins with densities similar to EVs. These drawbacks limit its efficiency in clinical settings.

Emerging methods like size exclusion chromatography, immunoaffinity capture, and polymer precipitation offer alternative approaches but also face challenges. These include cumbersome procedures, restrictions to specific protein targets, or contamination risks that can compromise subsequent analyses. Consequently, there is an urgent need for more efficient and standardized separation techniques in clinical diagnostics. Recent advancements offer promising alternatives. For example, Yin et al. developed peptide probes capable of capturing EVs by targeting their uniquely curved membranes and distinctive lipid profiles. This method, which surpasses traditional immunoaffinity techniques, focuses on the universal detection of phospholipids in EV membranes through peptide–lipid interactions, independent of protein and oligonucleotide content [60]. The study found that both monomeric and trimeric forms of bradykinin BK bind to synthetic nanovesicles and EVs, with polymerization enhancing the binding affinity to 7 μM. This approach represents a significant step forward in EV separation and detection, potentially improving clinical applications.

Additionally, several emerging technologies have been developed for the separation of EVs. One such technology is the ExoChip, a microfluidic affinity system that leverages both affinity binding and filtration. The ExoChip consists of microfluidic channels coated with a specific binding agent, such as an antibody targeting the EV surface marker CD63. As a sample containing EVs passes through the chip, the EVs are selectively captured by the binding agent, while other molecules are allowed to pass through. The ExoChip has shown high efficiency in isolating EVs, with a reported recovery rate exceeding 90% for four transmembrane proteins. It is also relatively fast, processing samples in <30 min, and offers a low-cost and user-friendly approach, making it a promising tool for EV isolation and analysis. Another widely used technology for EV isolation is Tangential flow filtration (TFF), also known as cross-flow filtration. TFF directs the fluid containing EVs tangentially across an ultrafiltration membrane, typically a hollow fiber membrane, rather than forcing it through the membrane. This method allows molecules smaller than the membrane’s molecular weight cutoff to pass through, while larger molecules, such as EVs, are retained and concentrated [61, 62]. TFF offers significant advantages over conventional filtration methods, such as reducing the risk of filter cake formation and pore clogging. Unlike size exclusion chromatography, which often dilutes EVs, TFF can concentrate them, making it particularly suitable for large-scale EV isolation from diluted samples. A specialized filtration technique known as standstill filtration dialysis[63] has also been developed. Using a 1000 kDa cellulose ester dialysis membrane, HFD allows for the separation of EVs from urine without the need for centrifugation, a common step in many ultrafiltration methods that can result in the loss of EVs.

Flow-field-flow fractionation (FFF) is an emerging technology for size-based separation and fractionation of EVs. The most prominent subtechnology within the FFF family is Asymmetric Flow-Field-Flow Fractionation (AsFlFFF), also known as AF4. A key advantage of AsFlFFF is its gentle fractionation process, which avoids the shear forces typically associated with stationary phases that can degrade particles, as seen in size exclusion chromatography. Moreover, AsFlFFF facilitates buffer exchange with EV formulation buffers, an important feature for therapeutic applications requiring the precise fractionation of EV subpopulations. In summary, the ongoing advancements in EV isolation technologies are paving the way for a new era in EV research, with significant potential to expand their clinical applications.

### The role and mechanism of action of EVs in tissue engineering and regenerative medicine

**Figure 2:**
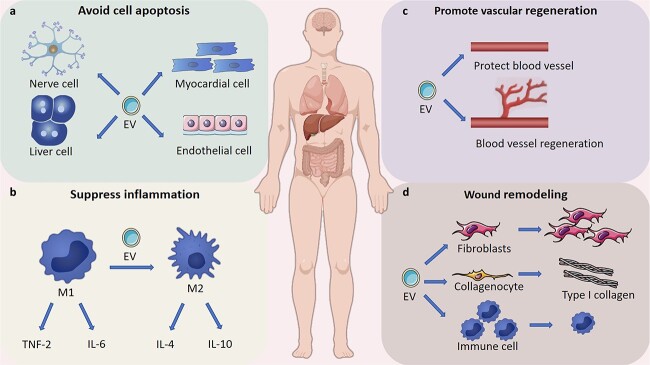
The role and mechanism of action of EVs in tissue engineering and regenerative medicine; (**a**) EVs prevent apoptosis and necroptosis of several cell types; (**b**) EVs stimulate macrophage polarization toward anti-inflammatory M2 phenotypes; (**c**) EVs promote vascular regeneration; (**d**) EV activates migration and proliferation of multiple cell types. *EVs* extracellular vesicles, *IL* interleukin

In recent years, EVs have garnered significant attention in tissue engineering and regenerative medicine due to their crucial roles in promoting cell proliferation, modulating immune response, enhancing vascular regeneration, and facilitating tissue remodeling (Figure 2). The underlying mechanisms driving these effects are intricate and multifaceted, highlighting the complexity of EV-mediated processes. As research continues to unravel these mechanisms, the potential clinical applications of EVs become increasingly evident, underscoring their value as therapeutic tools worth exploring and harnessing in future medical interventions.

Tissue injury can arise from various factors, including infections, autoimmune reactions, toxins, radiation, and trauma. Several critical processes are involved in the subsequent repair of damaged tissues: (1) inhibition of apoptosis and promotion of cell survival to minimize cell loss, (2) modulation of the immune response to prevent excessive inflammation, (3) angiogenesis, where endothelial cells migrate and proliferate to form new blood vessels, (4) cell proliferation and differentiation to replace lost or injured cells, and (5) regulation of fibrosis and ECM synthesis to control scar tissue formation. These immune-modulatory and tissue remodeling processes are significantly influenced by EVs from various differentiated cells, primarily stromal cells within the human body. Moreover, these processes can be further enhanced by EVs released from stem and progenitor cells [16].

#### Promotion of cell proliferation

EVs enhance cell proliferation through multiple mechanisms, including the regulation of signaling pathway, gene expression, modulation of the cellular microenvironment control of the cell cycle, and direct intercellular communication. EVs activate signaling pathways in target cells by delivering growth factors, hormones, proteins, and various types of RNA, which in turn promote cell proliferation. For instance, grape-derived EVs activate the Wnt/TCF4 signaling pathway to maintain intestinal homeostasis and stimulate cell proliferation [64]. Additionally, EVs influence the gene expression of target cells by transporting mRNAs, miRNAs, and other regulatory molecules, thereby either promoting or inhibiting cell proliferation. MSC-EVs, for example, promote skin cell proliferation and burn healing via the Wnt/β-catenin signaling pathway and encourage osteoblast differentiation through miRNAs such as miR-196a. Furthermore, EVs carry enzymes and other molecules that modify the microenvironment around cells, facilitating cell growth and division. An example of this is how tumor cell-derived EVs alter the behavior of surrounding normal cells, contributing to tumor growth and metastasis. EVs can also directly influence the cell cycle by delivering proteins and small molecules that regulate critical checkpoints, such as promoting the G1/S transition to advance the cell cycle. Finally, EVs function as a means of intercellular communication, capable of transmitting signals to distant cells through body fluids, thereby exerting systemic regulatory effects. As an emerging tool for intercellular communication, EVs play a vital role in regulating cell proliferation. Future research is crucial to further elucidate the precise mechanisms of EV action and to understand their specific functions in various physiological and pathological contexts.

#### Inhibiting inflammatory responses

EVs mitigate inflammatory responses and promote the differentiation of anti-inflammatory cells through various mechanisms, including immune modulation, suppression of inflammatory signals, intercellular communication, clearance of inflammatory mediators, and miRNA transfer. These actions contribute significantly to the treatment of inflammation-related diseases and the repair of tissue damage. First, EVs reduce inflammation by transporting immunomodulatory proteins or RNA molecules that regulate immune system activity and suppress the release of inflammatory mediators. For instance, a 2021 study by Sun *et al.* [65] demonstrated the potential of EVs in modulating inflammation by targeting inflammation-associated miRNAs, specifically miR-155, to reduce peripheral inflammation and monocyte/macrophage migration following HIV infection. Although EVs did not directly lower the HIV viral load, they showed promise in reducing neuroinflammation and regulating immune responses in HIV-infected mouse models by antagonizing miR-155. In addition, proteins and miRNAs carried by EVs can inhibit inflammatory signaling pathways, such as the PI3K/Akt-mTOR pathway and MyD88-NF-κB pathway, thereby suppressing inflammatory response and promoting the differentiation of anti-inflammatory cells. This has implications for the treatment of inflammation-related diseases and tissue repair. EVs also play a crucial role in intercellular communication, allowing them to influence the function and phenotype of target cells. For example, EVs derived from MSCs can modulate inflammatory responses by altering the polarization of T cells and macrophages and by selectively overexpressing specific miRNAs, such as miR-125b and miR-181a. Furthermore, EVs mitigate inflammation through the transfer of anti-inflammatory miRNAs, including miR-21, miR-23a, miR-125b, and miR-145. As key regulators of inflammatory responses, EVs have the potential to become novel therapeutic targets for treating inflammatory diseases. Their natural ability to serve as drug delivery systems can be harnessed to improve the targeting and stability of anti-inflammatory drugs. Moreover, the specific molecules encapsulated by EVs could serve as diagnostic and prognostic markers for inflammatory diseases, aiding in clinical decision-making. Continued research into the role of EVs in inflammation regulation is expected to provide new insights and strategies for treating inflammatory conditions and advancing more effective clinical therapies.

#### Promotion of vascular regeneration

EVs play a crucial role in promoting new blood vessel formation and tissue repair by carrying proangiogenic factors, activating specific signaling pathways, and transmitting miRNAs essential for tissue and organ regeneration, including the heart and skin. Endothelial cell proliferation and migration are key processes in vascular regeneration, contributing to blood vessels repair after injury and the formation of new vessels. EVs directly target vascular endothelial cells by delivering molecules such as growth factors, cytokines, and miRNAs, which promote endothelial cell proliferation, migration, and differentiation, thereby facilitating vascular regeneration and repair. For instance, Huang *et al.* [66] employed nanoflow cytometry to detect CD147 expression in plasma-derived small EVs (sEVs) from 155 hepatocellular carcinoma (HCC) patients, 59 cirrhosis patients, and 82 healthy donors. The study found that HCC-derived sEV-associated CD147 acts as a diagnostic marker and promotes endothelial cell angiogenesis through the PI3K/Akt pathway. In addition, microvesicles from other sources, such as endothelial progenitor cells (EPCs), human umbilical cord MSCs, and ADSCs, have been shown to activate signaling pathways like Wnt/β-catenin, AKT, and Sonic Hedgehog/RhoA, as well as regulate specific miRNAs, such as miRNA-31, to enhance vascular regeneration. Moreover, EVs serve as intercellular communication mediators, facilitating signal exchange between endothelial cells and surrounding support cells (such as smooth muscle cells and pericytes). This interaction regulates blood vessel stability and function, which is vital for vascular regeneration and repair. Additionally, EVs modulate the immune response and exert anti-inflammatory effects, creating a microenvironment conducive to vascular regeneration, offering significant potential for application in regenerative medicine and tissue engineering. The transition of EV applications in vascular regeneration from basic research to clinical practice is currently underway, with EVs poised to become essential tools for treating cardiovascular diseases and supporting tissue repair and organ regeneration in the future.

#### Facilitation of organizational reinvention

EVs play a crucial role in tissue remodeling and repair, including wound healing, organ regeneration, and managing pathological tissue alterations. Beyond promoting cell proliferation, reducing inflammation, and fostering vascular regeneration, EVs contribute to the repair and regeneration of damaged tissues by regulating cell differentiation and remodeling the ECM, among other mechanisms. First, EVs transport signaling molecules and transcription factors that influence cell fate, thereby significantly impacting cell differentiation. In processes like bone and cartilage remodeling or heart repair, EVs deliver signals that guide the differentiation of stem or progenitor cells into specific cell types. Additionally, EVs can selectively encapsulate a diverse array of proteins, lipids, and nucleic acids, packaged by stem cells in response to microenvironmental changes. For example, during COVID-19, SARS-CoV-2 infection can lead to lung inflammation and damage, but EVs released from injured alveolar epithelial cells can modulate the inflammatory response and carry repair signals to neighboring cells, promoting post-injury repair by regulating cell differentiation [67]. Furthermore, EVs play a vital role in tissue remodeling by interacting with the ECM. They carry enzymes such as metalloproteinases that participate in ECM degradation, thereby facilitating the deposition and remodeling of new substrates. Simultaneously, EVs can activate fibroblasts by delivering signaling molecules like TGF-β1 mRNA, promoting the production of a temporary ECM, which is essential for subsequent stages of tissue repair and regeneration. Currently, EVs hold significant potential in tissue engineering and regenerative medicine, though their mechanisms of action are complex and involve the regulation of various biomolecules and signaling pathways. Continued research into these mechanisms and the development of related clinical applications could provide new strategies and methods for treating a wide range of tissue injuries and diseases.

### EV engineering strategies in tissue engineering and regenerative medicine

**Figure 3:**
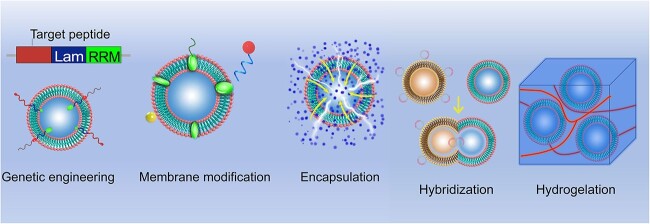
EV engineering strategies in tissue engineering and regenerative medicine. The performance of EVs can be improved through genetic engineering, membrane modification, encapsulation, hybridization, and hydrogelation. *RRM* RNA recognition motif, *EVs* extracellular vesicles

Native EVs have inherent functional limitations that necessitate optimization for improved targeting, controlled release, and functional integration. Engineered EVs, however, can be precisely tailored to enhance their therapeutic potential through various advanced techniques, including genetic engineering, surface modification, encapsulation, hybridization, and hydrogel incorporation **(**Figure 3**).** These innovations significantly boost the efficacy and versatility of EV-based therapies.

One approach to improving EV targeting involves genetic engineering. A landmark study by Alvarez-Erviti *et al.* [29] demonstrated this by engineering exosomes for specific small interfering RNA (siRNA) delivery. They genetically fused Lamp2b, an EV-enriched surface protein, with short peptides derived from rabies virus glycoprotein (RVG) or muscle-specific peptide, thereby directing the EVs to the brain or muscle tissue, respectively. Similarly, Delcayre *et al.* [68] developed the “exosome display technique,” which involves fusing the targeting moiety with the C1C2 domain of lactoadhesin to embed it on the EV surface, enhancing targeted delivery capabilities.

The lipid and protein composition of EV membranes enables a variety of surface modification techniques, such as lipid insertion, chemical ligation, enzymatic ligation, affinity binding, and metabolic labeling. These modifications can be achieved by incorporating lipid fragments into EV membranes through simple mixing and incubation, a method that is straightforward, cost-effective, and compatible with nearly all types of EVs without altering their morphology or biological properties. In contrast, chemical ligation methods, which utilize reactive groups on vesicle membrane lipids or proteins, enable the attachment of targeting peptides to EV surfaces through bio-orthogonal reactions. However, this approach can be nonspecific and may interfere with protein–protein interactions or alter EV properties [69].

Hybridization techniques represent another strategy for enhancing EV functionality. These involve combining isolated EVs with lipid nanoparticles to form hybrid particles with enhanced properties. A basic method of hybridization is incubation [70]. For instance, in osteoarthritis treatment, researchers have developed a hybrid chondrocyte affinity peptide exosome (CAP-Exo) by fusing the N-terminal gene of the chondrocyte exosome surface protein Lamp2b with a CAP. This hybrid EV, designed to encapsulate the CRISPR/Cas9 plasmid, is administered intra-articularly to prevent cartilage degradation and alleviate OA symptoms [71]. Such hybridization techniques enable the formation of nanovesicles that retain the surface properties of exosomes while accommodating larger therapeutic molecules [72].

Tissue engineering encompasses the integration of biomaterials, seed cells, and extracellular signaling molecules to effectively replace or regenerate severely damaged tissues. This interdisciplinary approach requires the synthesis of cell biology, materials science, and biomedical engineering principles [28]. EVs, as a pivotal component in tissue regeneration, must be delivered via systems that adapt to various materials and functional needs [73]. Biomaterials used in TE should conform to the irregular shapes of defects, offer fluidity, and adhere to surrounding tissues to ensure complete coverage and protect the healing site from external exposure. Hydrogels, which are water-swollen polymeric materials, have emerged as key players in this field. Their inherent versatility allows for the incorporation of different components to enhance the properties of nanoparticles, including EVs [28]. Hydrogels can be delivered through various methods, such as injection, and are commonly used due to their biocompatibility and ease of application [28]. They are typically made from natural water-soluble polymers, including amylopectin, chitosan, fibroin, cellulose, alginate, gelatin, and hyaluronic acid [73]. By manipulating substrate degradation rates or altering the polymer network structure, hydrogels can facilitate the controlled release of EVs, which is crucial for sustained therapeutic effects [28]. EVs, which carry proteins, RNA, DNA, and cytokines from parent cells, play a significant role in guiding target cell behavior, promoting stability, and minimizing immunogenicity. Various engineering techniques can further load EVs with therapeutic agents for specific purposes, making stem cell-derived EVs a superior option for regenerative therapies compared with direct stem cell use [73]. For example, Liu and colleagues (2017) combined imine-crosslinked hydrogels with human-induced pluripotent stem cell (hiPSC)-derived EVs to investigate their effects on articular cartilage repair [171]. Their study demonstrated that the gradual release of EVs over 14 days, facilitated by the hydrogel degradation, enhanced cellular regulation and promoted cartilage repair both *in vitro* and *in vivo*. Similarly, Shi *et al.* [74] showed that exosomes derived from GMSCs and incorporated into chitosan/silk hydrogels improved skin wound healing in diabetic rats by increasing re-epithelialization, inducing angiogenesis, and remodeling collagen. Furthermore, Chew *et al.* [76] recently reported that MSC-derived exosomes combined with collagen sponges enhanced periodontal regeneration in a rat model of periodontal defects. These advancements underscore the potential of EV-hydrogel systems in advancing tissue engineering and regenerative medicine.

Regenerative medicine focuses on curing diseases and facilitating the reconstruction of deformities and traumas through various innovative approaches. These methods include *in vivo* stem cell or biomolecule transplantation, the replacement of organs or tissues with cellular structures grown outside the body, and the use of biologically active biomaterials to enhance innate regenerative processes and restore organ or tissue function [77]. A particularly promising advancement in regenerative medicine is three-dimensional (3D) bioprinting, which integrates 3D printing technology with biological processes. This cutting-edge technique uses computer-aided design to pattern 3D cell and tissue structures within bioinks, involving the precise deposition of materials that contain cells, growth factors, and other bioactive molecules to create tissue-like constructs that emulate the properties of natural tissues. Bioinks can be derived from natural materials such as collagen, fibrin, hyaluronic acid (HA), agarose, silk, glycerin, cellulose, and alginate, or from synthetic materials often used in hydrogel form [78]. For instance, extrusion-based bioprinting technology, combined with biolinks like polycaprolactone, poly(lactic acid), poly(glycolic acid), and poly(ethanoic acid) (PEG), has enabled the creation of cellular human heart structures using patient cell-free omental tissues and cardiomyocytes. Additionally, Chen et al. utilized BMSC-derived EVs within a cartilage ECM/gelatin methacrylate hydrogel to produce a bioscaffold via stereolithography-based 3D printer. Their study demonstrated that EV-modified scaffolds significantly enhanced cartilage regeneration in animal models [79]. Nucleic acid-functionalized EVs are also showing great promise in regenerative medicine due to their advantages. For example, Mathiyalagan *et al.* [80] reported that EVs derived from CD34 stem cells can deliver miRNA precursors to target cells, effectively regulating gene expression. Similarly, Guo *et al.* [81] loaded MSC-derived exosomes with PTEN siRNA to facilitate SCI repair, highlighting the potential of MSC-derived exosomes to offer protective effects in various diseases, such as myocardial infarction [82], bone defects [83], and kidney diseases [84], and their ability to work synergistically with siRNAs in tissue repair. Yang *et al.* [85] developed a method for the large-scale production of mRNA-encapsulated EVs using a custom electroporation device, and recent studies have shown that EVs can deliver nerve growth factor (NGF) mRNA and protein effectively, potentially treating ischemic brain injuries. These advancements underscore the potential of nucleic acid-functionalized EVs as delivery vectors in gene therapy and their growing role in the biomedical field [33].

### Tissue-specific applications of engineered EVs in tissue engineering and regenerative medicine

Due to the limited efficiency and lack of targeted delivery of native EVs, modifications are often necessary to enhance their therapeutic potential. EEVs offer significant promise for tissue-specific applications by allowing for targeted delivery and improved treatment outcomes while minimizing side effects. First, surface modifications of EVs, such as the attachment of specific ligands or antibodies, can enhance their ability to accumulate in target tissues, thereby improving the precision and efficacy of therapeutic interventions. Second, internal modifications, including the encapsulation of specific drugs or genetic material, enable the regulation of therapeutic mechanisms directly within target tissues. This facilitates more precise and effective therapies. Additionally, altering the physical properties of EVs—such as their size and charge—can further optimize their distribution and functionality within the body. Ongoing research is focused on enhancing EV design to carry a range of therapeutic agents, including drugs, antibodies, proteins, and RNA. In this section, we present an overview of the latest research advances in this area, integrating information on disease types, engineering strategies, vesicle sources, and their respective advantages and disadvantages [86–105] (Table 1). Our goal is to offer beginners a clear understanding by analyzing key examples.

**Table 1:** The application of engineered EVs in disease treatment

EVs origin	Particle size	Disease	EVs engineering strategy	Functional mechanism	Merits and demerits	Refs.
*Apis mellifera* royal jelly	<150 nm	wound treatment	integrate RJ EVs into hydrogelation	antibacterial and biofilm-inhibiting properties and stimulated migration in MSCs	stimulates wound fibroblast deposition and tissue collagen, recruits wound-associated immune cells, and has not yet been developed as an ideal local delivery matrix for RJ EVs	[86]
BMSC	50–150 nm	Diseases of the spine	A novel injectable hydrogel was synthesized and combined with β-TCP to form PG/TCP (PEGMC with β-TCP)	Topical administration	The composite hydrogel improves the local microenvironment and exhibits better mechanical strength.	[87]
ADSC	10–30 μm	diabetic wound healing	ADSC-Exo was loaded into matrix metalloproteinase degradable polyethylene glycol (MMP-PEG) smart hydrogel	target the MMP2 response and significantly promote the recovery of diabetic wounds	Optimizing cell function to accelerate wound repair via AKT and ERK Pathways Limited clinical applications	[88]
BMSC	30–150 nm	SCI	3D gelatin methacrylate hydrogel (GelMA) is used as an Exo delivery system for transplantation (GelMA-Exos)	EV-loaded injectable hydrogel for minimally invasive treatment of spinal cord injury	less invasively into the damaged lesions to induce neurological functional recovery high biocompatibility	[89]
MSC	172–208 nm	chronic liver failure	PEG macromolecules are blended with MSC-EV to form EV-encapsulated PEG hydrogels	EV-loaded hydrogel based on click reaction has potential for systemic drug delivery	therapeutic effect on histological characteristics reversion of fibrosis and apoptosis	[90]
ADSC	95.82–122.12 nm	diabetic wound healing and skin regeneration	incorporation of ADSC- Exos into ECM hydrogel to form novel ECM hydrogel@exosomes (ECM@ Exos)	ECM hydrogel displays good biocompatibility and biodegradability.	effectively reduces inflammation and promotes angiogenesis, collagen deposition, cell proliferation, and migration, thereby accelerating the wound healing process	[91]
*A. mellifera* royal jelly	100 nm	Wound healing	A simple physical mixture of RJ-EVs and methacrylic anhydride-modified silk gum (SerMA)	SerMA/RJ-EVs hydrogel dressing has a porous internal structure and high fluidity.	SerMA/RJ-EVs hydrogel dressing offers a simple, safe and robust strategy for modulating inflammation and vascular impairment for accelerated wound healing.	[20]
CSF	20–300 nm	SCI	CSF-EVs were mixed with hydrogels and placed on the surface of the spinal cord.	CSF-EVs could enhance vascular regeneration by activating the PI3K/AKT pathway, hence improving motor function recovery after SCI	may offer potential novel therapeutic options for acute SCI; significantly improved the migration capacity of bEND.3 ​cells increased endothelial cell proliferation and angiogenic activities.	[92]
platelet membrane-engineered EVs	139.3–140.5 nm	IHD	Platelet mimic EVs (P-EVs) are prepared by extrusion by fusing the membrane of EVs with the platelet membrane.	membrane fusion, targeted delivery, EEVs with platelet membrane decoration	the adhesive proteins and natural targeting ability to injured vasculature of platelets retained and enhanced the pro-angiogenic potential of EVs	[93]
USC	40–100 nm	SCI	USC-Exo was embedded in a hydrogel and SCI treatment was performed by local injection	ANGPTL3 is enriched promote angiogenesis promote the angiogenic activity	enhancing spinal cord neurological functional healing reducing inflammation	[94]
HSC	50–200 nm	Liver	Cas9 RNP was loaded into purified exosomes isolated from hepatic stellate cells by electroporation.	The genome-editing delivery system efficient intracellular delivery	efficient delivery of Cas9 RNP enable precision therapy of liver diseases	[95]
HFSC	95 nm	Hair loss	A detachable MN patch-mediated drug delivery system	Transdermal drug delivery continuous administration of HFSC activator UK5099 accelerates the activation of HF MSCs.	stable keratin hydrogel structure continuous and effective transfer of drugs improve treatment compliance	[96]
MSC	105.43–110.21 nm	RD	EVs isolated from MSCs were engineered, modified with cRGD peptides on their surface, and loaded with anakinra, an antagonist of IL-1 receptors.	Membrane fusion technology nanodelivery system inhibit microglial activation and protect photoreceptors from apoptosis *in vitro*	Enhance the targeting ability Good stability and specific targeting effects	[97]
MSCs SαV-NVs PLT-NVs	200 nm	Myocardial ischemia/reperfusion (I/R) damage	the genetic modification of the cell membrane to create modified NVs, followed by the fusion of individual NVs to form hNVs	inhibition of CD47-SIRPα interaction Promotes macrophage phagocytosis of dead cells	relieves inflammation of the heart muscle Minimize infarct size Improves I/R model cardiac function	[98]
MSCs Exocarpium Citri grandis	141 nm	Heart transplant rejection	Hybridized cell membrane vesicles loaded with anti-inflammatory drugs	FNV@RAPA Targets Heart Transplant Sites Effectively mitigates IRI Promotes macrophage polarization to anti-inflammatory phenotype	Alleviate early transplant complications and immune rejection	[99]
MSCs	25 nm	Rheumatoid arthritis	A cerium nanoparticle-fixed mesenchymal stem cell nanovesicle hybridization system	Clearance of excess ROS; modulation of immune cell phenotype; protection of chondrocytes	Ce-MSNVs provide immediate relief of inflammation, prevent bone destruction and modulate the immune microenvironment	[100]
ANeuM	280–320 nm	Acute Respiratory Distress Syndrome (ARDS)	Activated neutrophil membrane fusion lung tissue targeted lipids and therapeutic lipid formation engineered biomimetic nanovesicles (DHA@ANeu-DDAB)	Inhibits neutrophil recruitment Reduces inflammatory cytokines Promotes macrophage burial and inflammation resolution	Comprehensive and effective inhibition of the development of lung inflammation Promotes acute lung injury repair	[101]
PM HSCM	250 nm 240 nm	Liver fibrosis	Construction of a delivery system for biomaterial-loaded exosomes and melatonin	ERS in hepatic stellate cells improves liver fibrosis Improves OS and ERS in hepatocytes and reduces TGFβ secretion	It has a good therapeutic effect on liver fibrosis Good targeting and biosafety	[102]
Neutrophil membrane	158.2 ± 38.9 nm	Ischemic stroke	A neutrophil membrane-coated ROS-responsive polyprodrug nanoparticle	NRN-Mediated M2 Polarization of Microglia Suppresses Inflammation Effective in reducing or even preventing cardiovascular side effects	Significantly improved behavioral function and promoted neurological recovery in MCAO mice after ischemic stroke Reduces inflammation of the nerves	[103]
RBC	210 ± 2.5 nm	Vascular endothelial injury	A spontaneous right-sided outward-driven coupling-driven ROS-sensitive nanotherapy was constructed	It improves targeted accumulation of myocardial damage and enhances repair of damaged myocardium.	Repair damaged endothelial cells Restore vascular permeability and a low inflammatory microenvironment	[104]
EXPLOR-engineered EVs	149 nm	Intervertebral disc degeneration (IVDD).	Motion self-powered triboelectric reactive MN load engineered EVs detection system	MN reduces inflammatory senescence in NP cells Blocks nuclear translocation and eliminates cell membrane damage	It effectively alleviates the degradation of *in vitro* diagnostic equipment and provides a promising repair strategy for sports-related diseases	[105]
macrophage	100 nm	Type 1 Diabetes	Artificial extracellular vesicles with high expression of PD-L1 and Galectin-9 were prepared using engineered cell lines	Inhibits islet-specific T cell activity and induces apoptosis Promotes differentiation and formation of spleen-derived regulatory T cells	Good therapeutic effect Effectively alleviated hyperglycemia in neo-NOD mice Slows down T1D progression	[105]

*SCI* spinal cord injury, *MSCs* mesenchymal stem cells, *BM-MSCs* bone marrow MSCs, *GMSCs* gingival MSCs, *HF-MSCs* hair follicle MSCs, *hBMSCs* human BMSCs, *P-MSC* placental MSCs, *hiPSC-MSCs* human induced pluripotent stem cell-derived MSCs, *hucMSC* human umbilical cord MSC, *UMSCs* umbilical MSCs, *EVs* extracellular vesicles, *CSF-EVs* EVs from cerebrospinal fluid, *USC-Exos* urogenic stem cell exosomes, *ADSC-Exos* adipose-derived stem cell exosomes, *ADSC-EVs* adipose-derived stem cell EVs

#### Skin/wound healing

Tissue repair and regeneration unfold in three main phases: inflammation, repair, and remodeling. The initial inflammatory response is crucial for eliminating pathogens from the wound site, clearing damaged cells, and renewing the ECM [106, 107]. This phase sets the stage for transitioning to the repair and remodeling phases, where new tissue is formed. During the repair phase, key processes such as angiogenesis, fibroblast activation, and ECM deposition occur to support tissue regeneration and restore function [108, 109].

Hair follicle regeneration offers hope for treating hair loss, yet developing efficient and manageable treatment strategies remains challenging. A novel microneedle (MN) patch system made from hair-derived keratin has been designed for the sustained delivery of hair follicle stem cell (HFSC) activators [96]. This MN patch, combined with MSC-derived exosomes and the small molecule drug UK5099, significantly enhances treatment efficacy while reducing the required doses. In a mouse model, this transdermal delivery system facilitated pigmentation deposition and hair regrowth over two rounds of administration within 6 days, showing superior results compared with subcutaneous injection of exosomes and local administration of UK5099.

**Figure 4:**
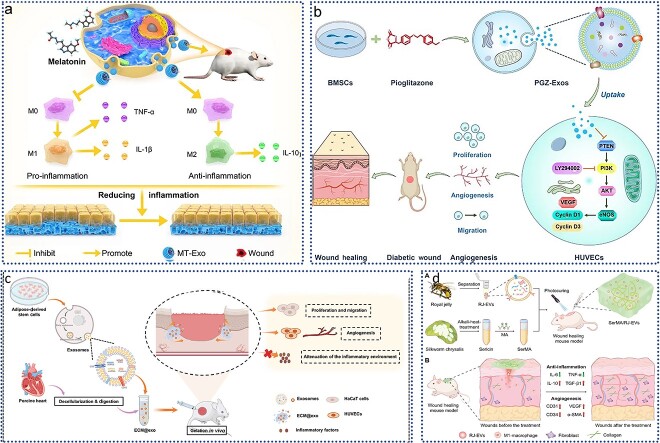
Application of EVs in wound healing. (**a**) MT-Exo promotes diabetic wound healing by modulating M1 and M2 macrophage polarization; adapted with permission from ref [110]. copyright 2020, the author(s); (**b**) PGZ-exos promotes the angiogenic function of HUVECs and accelerates diabetic wound healing; adapted with permission from ref [111]. copyright 2021, the author(s); (**c**) preparation of ECM@exos and the role of ECM@exos in the wound healing process; adapted with permission from ref. [91], copyright 2023, the author(s); (**d**) the SerMA/RJ-EV hydrogel modulates inflammation and vascular impairment to accelerate wound healing; adapted with permission from ref. [20], copyright 2023, the author(s). *MT-Exo* melatonin-pretreated MSCs-derived exosomes, *HUVECs* human umbilical vein vascular endothelial cells, *PGZ-exos* pioglitazone, *ECM@exo* extracellular matrix hydrogel@exosomes, *EVs* extracellular vesicles, *IL* interleukin

In diabetic patients, wound recovery can be hindered by prolonged inflammation, which often leads to poor healing outcomes. Addressing this issue has been challenging, but recent research has highlighted the potential of MSC-derived exosomes (Exo) for diabetic wound healing due to their anti-inflammatory properties. Liu *et al.* [110] investigated the enhanced effects of melatonin (MT)-pretreated MSC-derived exosomes (MT-Exo) on diabetic wound healing and elucidated their mechanisms. As illustrated in Figure 4a**,** MT-Exo were found to suppress inflammation by shifting the macrophage polarization from the pro-inflammatory M1 type to the anti-inflammatory M2 type through activation of the PTEN/AKT signaling pathway. This shift not only mitigates excessive inflammation but also promotes angiogenesis and collagen synthesis, thereby accelerating wound healing in diabetic conditions. These findings suggest that MT-Exo represent a promising strategy for improving diabetic wound healing and offer a potential therapeutic approach for enhancing recovery in diabetic patients.

Enhanced angiogenesis is crucial for promoting diabetic wound healing. Hu *et al.* [111] investigated the effects of pioglitazone-pretreated exosomes derived from MSCs (PGZ-Exos) on diabetic wound healing. Their study revealed that PGZ-Exos, derived from BMSCs treated with pioglitazone, significantly improved diabetic wound healing. As illustrated in Figure 4b, PGZ-Exos enhanced the biological functions of human umbilical vein endothelial cells (HUVECs), including their migration, tube formation, and wound repair capabilities, by activating the PI3K/AKT/eNOS signaling pathway. This activation promoted angiogenesis and accelerated wound healing in diabetic conditions. These findings highlight PGZ-Exos as a promising novel cell-free therapeutic approach for treating diabetic wounds, offering an innovative strategy to enhance wound recovery.

Conventional wound healing approaches often struggle with infection risks and limited therapeutic efficacy, presenting a significant clinical challenge in promoting effective and safe wound repair. Fortunately, engineered EVs offer promising new solutions for this issue. As depicted in Figure 4c, researchers developed an injectable, thermosensitive hydrogel by first creating ECM hydrogels from porcine left ventricular myocardium through decellularization and digestion. Exosomes derived from ADSCs were isolated by UC and then encapsulated within the ECM hydrogel, resulting in the ECM@Exos construct. This hydrogel releases ADSC-derived exosomes slowly and continuously, maintaining a high concentration at the wound site while gradually degrading *in vivo*. Both *in vitro* and *in vivo* studies demonstrated that ECM@Exos treatment effectively reduced inflammation and enhanced angiogenesis, collagen deposition, and cell proliferation and migration, thereby accelerating wound healing. This approach highlights the potential of exosome-based hydrogels in advancing wound care [91]. In addition to hydrogels, other biomaterials are also being explored for wound dressings. For instance, researchers developed a SerMA/RJ-EV hybrid hydrogel dressing by combining methacrylic anhydride-modified silk gel (SerMA) with royal jelly-derived EVs (RJ-EVs), as shown in Figure 4d. The RJ-EVs in the SerMA hydrogel demonstrated excellent slow-release properties *in vivo* and significantly accelerated wound healing in a full-thickness skin defect model by promoting cell proliferation and angiogenesis. RNA sequencing revealed that the SerMA/RJ-EV hydrogel dressing involved pathways related to inflammatory injury repair, including recombinational repair, epidermal development, and Wnt signaling. This dressing also proved effective in improving wound healing, suggesting its great potential for clinical applications in wound care and other diseases [20].

Effective skin wound healing requires a coordinated interplay of skin cell proliferation, migration, differentiation, and apoptosis. In addition to promoting cell proliferation, it is crucial to prevent excessive scarring due to myofibroblast aggregation. MSC-derived exosomes can activate various wound healing pathways, such as those involving Akt [110], ERK [112], and STAT3, and promote the expression of growth factors like HGF, IGF-1, NGF, and stromal cell-derived factor-1. The role of MSC-derived exosomes in skin tissue regeneration is dynamic and varies with cell density. For example, Zhang et al. demonstrated that exosomes derived from human umbilical cord mesenchymal stem cells (hucMSC-Exos) activate β-catenin and enhance skin stem cell proliferation in the early stages of cutaneous regeneration while limiting excessive cell expansion through the transfer of the 14–3–3ζ protein, which inhibits Wnt/β-catenin signaling [115]. Similarly, Fang *et al.* [113] found that exosomes from umbilical cord EVS promote wound healing and reduce scar formation and myofibroblasts aggregation. High-throughput RNA sequencing and functional analysis revealed that UMSC-EXO-specific miRNAs (miR-21, miR-23a, miR-125b, and miR-145) inhibit fibroblast differentiation into myofibroblasts by suppressing the TGF-β/SMAD2 signaling pathway.

#### Motor system

Bone injuries are prevalent in clinical settings, ranging from minor fractures to severe bone breaks and joint dislocations. These injuries not only inflict physical pain but can also impact patients’ daily lives and work abilities. MSCs are currently among the most promising candidates for bone tissue engineering and regeneration [114, 116]. Recent research has shifted focus from using the cells themselves to leveraging EVs secreted by these cells, based on the paracrine hypothesis. Furuta *et al.* [117] demonstrated that CD9-deficient mice with femur transverse fracture exhibited significantly impaired bone healing compared with wild-type controls. Treatment with MSC-derived exosomes and MSC-conditioned medium improved healing in CD9-deficient mice and further accelerated the healing process in wild-type mice, highlighting the potential of MSC-EVs to enhance bone repair.

**Figure 5:**
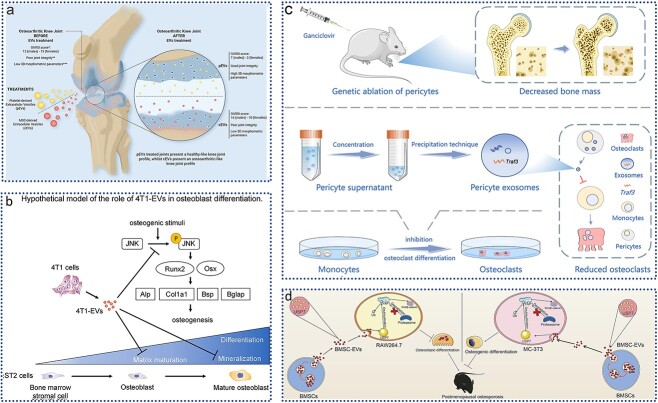
Application of EVs in motor system injury. (**a**) intra-articular injection of platelet lysate-derived EVs restored knee osteoarthritis in an *in vivo* rat model; adapted with permission from ref. [118], copyright 2024, the authors; (**b**) bone metastatic mammary tumor cell-derived EVs inhibit osteoblast maturation; adapted with permission from ref. [119], copyright 2023, Elsevier Inc. (**c**); perivascular cell-derived EVs inhibit bone resorption; adapted with permission from ref. [120], copyright 2023, the authors; (**d**) EVs from bone MSCs alleviate osteoporosis in mice; adapted with permission from ref. [121], copyright 2023, Elsevier Inc. *EVs* extracellular vesicles, *4T1-EVs* 4T1 mouse mammary tumor cell-derived EVs, *Runx2* runt-related transcription factor 2, *Osx* osterix, *alp* alkaline phosphatase, *Col1a1* collagen type I, *Bsp* bone sialoprotein, *JNK* c-Jun N-terminal kinase, *BMSCs* bone marrow-derived MSCs, *YAP1* Yes1 associated transcriptional regulator, *USP7* ubiquitin specific peptidase 7

Beyond MSCs, EVs from other cell types also contribute to bone tissue repair. As shown in Figure 5a researchers compared the regenerative potential of MSC-EVs (cEVs) with platelet-derived EVs in a rat model of osteoarthritic cartilage. Computed tomography analysis and OARSI scoring revealed that platelet-derived EVs have notable regenerative potential for osteoarthritic cartilage, with a particularly significant effect in female rats, showing superior efficacy compared with cEVs [118].

Tumor cell-derived EVs are known to promote osteoclast differentiation and bone resorption, yet their impact on osteoblast differentiation and function is less understood. Uehara *et al.* [119] investigated the effects of EVs from bone metastatic breast cancer cells on osteoblast activity *in vitro*. As depicted in Figure 5b, EVs from 4T1 mammary tumor cells derived from bone-metastatic mice (4T1-EVs) inhibited stromal mineralization in bone marrow stromal cells from ST2 mice under osteogenic conditions. Furthermore, 4T1-EVs significantly reduced levels of phosphorylated c-Jun N-terminal kinase (JNK), which were elevated under osteogenic induction. Analysis of osteoblast marker gene expression revealed a decrease in expression at later stages of osteoblast differentiation. These findings suggest that 4T1-EVs impair osteoblast maturation, likely through JNK regulation, providing new insights into the pathological effects of osteolytic bone metastases and the role of EVs in osteoblast differentiation. Similarly, there is an effect of EVs on osteoclast differentiation [120]. In addition, the ability of EVs to enhance osteogenesis allows them to be used as a promising tool for the treatment of bone damage repair [121].

Healing large bone defects remains a significant challenge in clinical practice, with successful outcomes depending heavily on promoting both angiogenesis and osteogenesis. Angiogenesis, the formation of new blood vessels, is essential as it precedes and supports osteogenesis, the process of bone formation. Developing artificial scaffolds that replicate the *in vivo* environment to enhance both angiogenesis and osteogenesis is critical for effective bone regeneration. This study presents a novel injectable dual-drug system utilizing a chitosan nanofiber microsphere-based poly (D, L-lactide-co-glycolide)-b-poly (ethylene glycol)-b-poly (D, L-lactide-co-glycolide) (PLGA-PEG-PLGA) hydrogel. This system incorporates VEGF for rapid angiogenesis and microspheres loaded with dental pulp stem cell-derived exosomes (DPSC-Exos) for sustained osteogenesis. The hydrogel effectively promoted angiogenesis in HUVECs and enhanced the osteogenic differentiation of preosteoblasts. When transplanted into a cranial bone defect *in vivo*, the hydrogel significantly facilitated bone formation, demonstrating a promising approach to improving bone regeneration by sequentially replicating the natural processes of angiogenesis and osteogenesis [122].

Osteoclasts and osteoblasts function synergistically in bone development and remodeling. EVs derived from pericytes also play a regulatory role in osteoclast differentiation. As shown in Figure 5c, researchers used microcomputed tomography and histological staining to assess changes in bone mass and osteoclast activity in mice lacking pericytes. Pericyte-derived EVs (PC-EVs) were collected and co-cultured with monocytes to observe their effect on osteoclast differentiation. RNA sequencing and protein blotting further elucidated the mechanisms involved. Results indicated that pericyte deletion led to increased bone resorption and reduced bone mass. PC-EVs inhibited the nuclear factor κ light chain enhancer (NF-κB) pathway in B cells through tumor necrosis factor receptor-associated factor 3 (Traf3), which negatively regulates osteoclast development and bone resorption. Silencing Traf3 in PC-EVs negated their inhibitory effect on osteoclast differentiation [120].

MSCs are recognized for their self-renewal and multidirectional differentiation potential [123]. MSCs and their derived EVs have emerged as promising tools for bone regeneration. As shown in Figure 5d, Wang *et al.* [121] investigated the effects of EVs from BMSCs (BMSC-EVs) in osteoporosis and their underlying mechanisms. In their study, EVs isolated from mouse BMSCs were used to treat an ovariectomy-induced osteoporosis model. Treatment with BMSC-EVs restored bone mass and strength, reduced trabecular bone loss and cartilage damage, and enhanced osteogenesis while inhibiting osteoclastogenesis in ovariectomized mice. *In vitro*, EVs improved osteogenic differentiation of MC-3 T3 cells and inhibited osteoclast differentiation of RAW264.7 cells. Ubiquitin-specific peptidase 7 delivered by BMSC-EVs stabilized Yes-associated protein 1 (YAP1), promoting bone formation via Wnt/β-catenin activation. Recently, bacteria-derived outer membrane vesicles (OMVs) have garnered significant attention in the field of bone regeneration. In a notable study, Zhou et al. developed a probiotic-derived OMV coating that enhances bone tissue repair by utilizing ultrasound for anti-infection purposes and leveraging OMV-mediated immune modulation [124].

Engineered EVs offer innovative approaches for bone tissue repair. Conventional bone grafts present challenges, and bioactive materials offer an alternative for bone repair and regeneration. Researchers have explored the osteogenic potential of β-tricalcium phosphate (B-TCP) loaded with hiPSC-MSC-derived EVs (hiPSC-MSC-Exos) for repairing critical-size cranial defects in deovulated rats. Histological analyses revealed minimal bone formation with β-TCP alone, whereas hiPSC-MSC-Exos significantly enhanced bone formation, with the scaffold covering most of the defect area, thus markedly improving osteogenesis [125]. Similarly, Qin et al. demonstrated the osteogenic capabilities of BMSC-EV-loaded hydrogels in repairing critical-sized skull bone defects [125, 126].

#### Circulatory system

EVs play a crucial role in various physiological processes, including the restoration of blood perfusion, delivery of factors to injury sites, and promotion of functional recovery and tissue regeneration. Therapeutic effects of EVs are partly attributed to their ability to enhance angiogenesis at injury sites, such as in skin wound healing [9], bone regeneration, ischemic limb recovery, and vascular injury repair [127]. Research demonstrates that EVs can influence key angiogenic processes, including endothelial cell proliferation, migration, and tube formation, as well as affect gene expression and protein secretion related to angiogenesis. Stimulating angiogenesis through the application of exogenous EVs represents a promising strategy for treating a range of diseases. However, translating EV-mediated angiogenesis into clinical practice requires further investigation into the underlying mechanisms and the identification of key regulators of angiogenesis.

Rapid reendothelialization after vascular injury is critical for maintaining endothelial integrity and preventing vascular disease. Recent studies have highlighted that transient implantation of exogenous EPCs can enhance the regeneration of damaged vessels through paracrine signaling [128]. For example, exosomes derived from human umbilical cord blood-derived endothelial cells were shown to promote vascular regeneration in a balloon-injured rat carotid artery model. Compared with phosphate-buffered saline, EPC-derived exosomes (EPC-Exos) significantly reduced intimal hyperplasia and accelerated reendothelialization at 14 and 21 days post-injury. Additionally, EPC-Exos enhanced the proliferation and migration of human microvascular endothelial cells and increased the expression of angiogenesis-related genes *in vitro* [129]. In the context of limb ischemia, which results from inadequate blood perfusion due to vascular lesions, multiple injections of exosomes from human induced pluripotent stem cell-derived mesenchymal stem cells (hiPSC-MSCs) have shown promise in stimulating blood perfusion and functional recovery of ischemic limbs. These exosomes also increased mean microvessel density in ischemic muscle and enhanced endothelial cell migration, proliferation, and tube formation. The expression of angiogenesis-related genes and proteins was significantly upregulated in endothelial cells [130]. Similarly, the injection of human MSC-derived microvesicles significantly improved blood perfusion rates in a rat hindlimb ischemia model after 2 weeks of treatment under hypoxic conditions [131].

**Figure 6:**
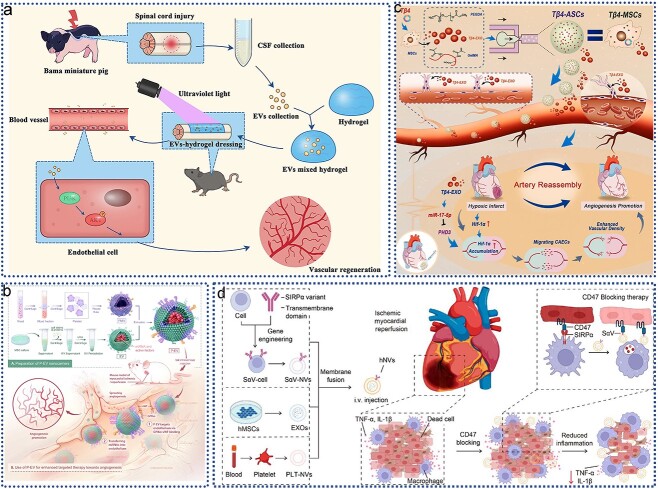
Application of EVs in circulatory system injury. (**a**) cerebrospinal fluid-derived EVs promote vascular regeneration through the PI3K/AKT signaling pathway; adapted with permission from ref. [92], copyright 2023, the authors; (**b**) schematic diagram of P-EV fabrication and targeted therapy for angiogenesis; adapted with permission from ref. [93], copyright 2021, the authors; (**c**) schematic diagram of the assembly of engineered artificial stem cells (Tβ4-ASCs) and their cardioprotective effects on the sustained release of Tβ4 exosomes in myocardial infarction; adapted with permission from ref. [132], copyright 2022, the authors; (**d**) schematic of hNVs with genetic editing enhance phagocytosis and mitigate inflammation for the I/R; adapted with permission from ref. [98], copyright 2024, American Chemical Society. *EVs* extracellular vesicles, *IL* interleukin *hNVs* hybrid nanovesicles, *SαV-NVs* SIRPα variants, *MSCs* mesenchymal stem cells, *PLT-NVs* platelet-derived nanovesicles

EVs from cerebrospinal fluid (CSF-EVs) have shown promising effects on motor function recovery following SCI. As illustrated in Figure 6a, researchers extracted cerebrospinal fluid from Parma miniature pigs and utilized UC to isolate the EVs. These CSF-EVs were then attached to a hydrogel and applied to the spinal cord surface. *In vivo* experiments demonstrated that vascular endothelial cells were capable of taking up CSF-EVs, and CSF-EVs from SCI pigs (SCI-EVs) were more effective in promoting angiogenesis compared with those from pigs that underwent laminectomy (sham-EVs). This study indicates that CSF-EVs can enhance angiogenesis via the activation of the PI3K/AKT pathway, potentially offering a novel therapeutic approach for acute SCI [92].

Platelet membrane-engineered endothelial cells may also improve therapeutic angiogenesis for ischemic heart disease. As depicted in Figure 6b, platelet-mimicking EVs (P-EVs) were created using an extrusion method, and a mouse myocardial ischemia/reperfusion (MI/R) model was used to evaluate their targeting and angiogenic effects. The study found that P-EVs, inheriting adhesion proteins and natural targeting abilities from platelets, selectively accumulated in the injured platelet vascular system and retained the proangiogenic potential of EVs. In the MI/R model, P-EVs preferentially targeted damaged endothelial cells in the ischemic heart, enhancing the angiogenic efficacy of EVs. This membrane fusion engineering strategy endows EVs with platelet targeting capabilities, presenting exciting prospects for designing other targeted EVs fused with different cell membranes for therapeutic angiogenesis [93].

Engineered exosomes, capable of regulating gene expression related to angiogenesis in target organs by delivering biologically active substances such as miRNAs, face challenges in promoting sustained angiogenesis due to their rapid and uncontrollable release at sites of myocardial infarction. Encapsulation in hydrogels may offer a solution for continuous release, but these hydrogels often have rapid release rates and high viscosity, complicating injection. In response, microspheres prepared via microfluidics have gained attention for their multiscale structure, functional properties, and injectability. These microspheres are already used in drug delivery and can sustain the release of therapeutic agents like NGB for up to 5 weeks. As shown in Figure 6c, Chen *et al.* [132] developed artificial stem cells capable of continuously releasing Tβ4 exosomes (Tβ4-ASCs) by encapsulating them in microspheres using microfluidics. Their results indicated that Tβ4-ASCs significantly enhanced peripheral coronary artery collateralization in myocardial infarction areas, outperforming direct exosome injections. Additionally, Tβ4-ASC-derived exosomes improved the angiogenic potential of coronary artery endothelial cells through the miR-17-5p/PHD3/Hif-1α pathway. This approach highlights the potential of Tβ4-ASCs as functional exosome carriers to stimulate collateral circulation formation after myocardial infarction, offering a viable clinical alternative.

Beyond microspheres, magnetic nanoparticles also show promise as exosome carriers. Researchers improved angiogenesis and cardiac function in infarcted tissues using magnetic nanoparticles to capture exosomes locally from circulation. These nanoparticles, consisting of an iron tetraoxide (Fe3O4) core and a silica shell modified with PEG, bind to antibodies targeting the CD63 antigen on exosomes or the myosin light chain on damaged cardiomyocytes. Under a local magnetic field and acidic pH conditions, the nanoparticles release the captured exosomes. In rabbit and rat myocardial infarction models, magnetic guidance of CD63-expressing exosomes led to their accumulation in infarcted tissues, reducing infarct size and improving left ventricular ejection fraction and angiogenesis. This method offers a way to control the biodistribution of endogenous exosomes for treating various diseases [133]. In a recent study, Rao et al. developed a novel cell membrane bionic vesicle for repairing myocardial ischemia/reperfusion injury (Figure 6d). 10.1021/acsnano.3c10784 This study utilized cell-derived nanovesicles from three different sources to create genetically engineered hybrid nanovesicles (hNVs). These hNVs demonstrated the ability to effectively clear dead cells and suppress inflammatory responses, thereby facilitating cardiac repair. This provides a new idea for immunotherapy of tissue damage diseases [98].

#### Nervous system

Neural tissue engineering, nanotechnology, and neuroregeneration are three distinct yet interconnected biomedical disciplines dedicated to addressing the intricate challenges associated with central nervous system (CNS) repair. The CNS is known for its limited regenerative capacity due to its microenvironment, which impedes effective healing and complicates the development of therapeutic strategies. Given the high incidence of CNS disorders such as stroke, which can severely impact brain function and pose a substantial burden on individuals and healthcare systems, identifying optimal treatment methods to restore functionality to pre-injury levels is crucial. Among the promising approaches explored, EVs derived from MSCs have emerged as a notable option. These EVs facilitate recovery by enhancing intercellular communication at the nanoscale while mitigating the risks associated with direct stem cell transplantation. Concurrently, advances in tissue engineering and regenerative medicine have introduced the use of hydrogels as biological scaffolds, providing essential matrix support for neural repair and enabling the controlled release of biomolecules that promote healing. The integration of sustained EV delivery with hydrogels’ structural support and guidance cues offers a promising strategy for neural tissue remodeling and regeneration.

Genetic engineering approaches can further enhance the therapeutic potential of EVs by enriching parental cells with specific biomolecules such as proteins[134] and RNAs (e.g. miRNAs or siRNAs). For instance, Li et al. demonstrated that MSCs transfected with the miR-133b gene produced EVs with ~2.5 times higher levels of miR-133b compared with controls, leading to improved functional recovery in an SCI model [133]. Additionally, Casella’s research group achieved significant anti-inflammatory effects in source cell-derived EVs by introducing a plasmid encoding the anti-inflammatory cytokine IL-4. These transgenic EVs, featuring surface overexpression of the “eat-me” signal lactate mucin (Mfg-e8), were able to target phagocytes in the brain effectively, resulting in marked reductions in neuroinflammation and autoimmune encephalomyelitis in a mouse model of multiple sclerosis [135].

Retinal degeneration (RD) is an irreversible condition leading to blindness, severely affecting patients’ quality of life and mental well-being. Targeting and regulating overactivated microglia is a promising strategy for managing RD. While MSC transplantation has shown potential due to its immunomodulatory and regenerative properties, challenges such as poor cell migration and integration persist. Recent study aimed at overcoming these obstacles by developing a nanodelivery system to specifically target overactivated microglia, inhibit proinflammatory factor release, and provide sustained neuroprotection. This approach involves EEVs derived from MSCs by attaching cyclic RGD (cRGD) peptides to their surface and loading them with the interleukin-1 receptor antagonist anakinra. Engineered cRGD-EVs demonstrated superior targeting efficiency against overactivated microglia and offered effective protection to photoreceptors in both experimental RD cells and animal models. This strategy enhances drug delivery to degenerating retinas and represents a promising method for treating RD by targeting and regulating the immune microenvironment [97].

**Figure 7:**
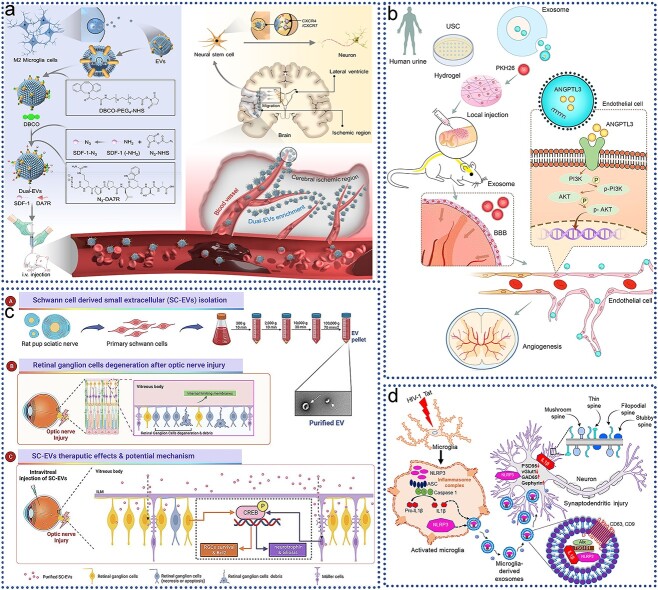
Application of EVs in nervous system injury. (**a**) schematic illustration of the effects of DA7R-SDF-1-EV (dual-EV) treatment on ischemic stroke; adapted with permission from ref. [136], copyright 2023, Chinese Pharmaceutical Association and Institute of Materia Medica, Chinese Academy of Medical Sciences; (**b**) human USC-derived exosomes ANGPTL3 mediates functional recovery after spinal cord injury by promoting angiogenesis; adapted with permission from ref. [94], copyright 2021, the authors; (**c**) Schwann cell-derived EVs as potential treatments for RGC degeneration. Adapted with permission from ref. [137], copyright 2023, the authors; (**d**) schematic diagram showing the involvement of microglial NLRP3 in exosome-induced synaptic dendrite damage in neurons; adapted with permission from ref. [138], copyright 2022, the authors. *UCS* urogenic stem cell, *EVs* extracellular vesicles

EVs not only affect neuronal differentiation at the damaged site [136], but are also able to have protective and regenerative effects on the optic nerve [137]. However, not all EVs have beneficial effects on the nerves, and some can also lead to impaired neuronal transmission [138]. As illustrated in Figure 7a, this study revealed that EVs secreted by M2 microglia promote the differentiation of neural stem cells (NSCs) at the injury site [136], consistent with recent findings that these EVs can enhance NSC differentiation in damaged tissues [139]. The modification of EVs with injury vessel-targeting peptide (DA7R) and stem cell recruiting factor (SDF-1) through copper-free click chemistry allows these Dual-EVs to recruit NSCs, induce their neuronal differentiation, and function as nanocarriers for the injured area. These Dual-EVs demonstrated the ability to target HUVECs *in vitro*, recruit NSCs, and promote their neuronal differentiation. *In vivo* experiments revealed that Dual-EVs could accumulate in the ischemic regions of stroke model mice, facilitate NSC recruitment, and enhance neurogenesis [136].

SCI remains a severe clinical challenge due to the lack of effective treatments. Researchers have been investigating the potential of implanting human urogenic stem cell exosomes (USC-Exos) within hydrogels to enhance functional recovery following SCI and to understand the underlying mechanisms. As shown in Figure 7b, studies have evaluated how USC-Exos influence functional recovery and examined the role of ANGPTL3, a protein found in USC-Exos, in promoting angiogenesis in SCI models. Results revealed that USC-Exos, when locally injected and encapsulated in hydrogels, successfully crossed the blood-spinal cord barrier and delivered ANGPTL3 to the injury site. This delivery facilitated spinal cord neurological recovery by promoting angiogenesis. Mechanistic investigations indicated that ANGPTL3, enriched in USC-Exos, was crucial for their angiogenic effects, mediated through the PI3K/AKT signaling pathway. Thus, USC-Exos, by delivering ANGPTL3, show promise as a novel therapeutic agent for spinal cord repair. Additionally, conductive hydrogels, which mimic the electrophysiological and mechanical properties of nerve tissue, are ideal for SCI repair [94]. Fan *et al.* [140] developed a conductive hydrogel incorporating BMSC-EVs within a network of UV-crosslinked methacrylated gelatin units. The hydrogel was treated with polypyrrole (PPy), tannic acid, and ammonium persulfate to enable *in situ* polymerization and crosslinking of conductive PPy chains. This hydrogel, loaded with BMSC-EVs, effectively inhibited inflammation, enhanced NSCs recruitment, and promoted neuron and myelin-associated axon regeneration. In contrast, Han *et al.* [141] designed a GelMA hydrogel-based novel MN patch for targeted delivery to the injured spinal cord. This approach also promoted angiogenesis, reduced fibrotic scar formation, enhanced NSC recruitment and differentiation, inhibited inflammation and oxidative stress, and supported neuronal cell regeneration. However, it is important to consider that hydrogels can cause excessive swelling and increase intracranial pressure, which could heighten the risk of secondary injury in CNS applications [142].

Optic neuropathy, characterized by the progressive degeneration of retinal ganglion cells (RGCs), is a leading cause of irreversible blindness. Schwann cell transplantation has emerged as a potential treatment, with evidence suggesting that SCs exert their effects through paracrine mechanisms. As shown in Figure 7c, Zhu *et al.* [137] investigated the neuroprotective and regenerative effects of Schwann cell-derived EVs (SC-EVs) following optic nerve injury. SC-EVs were internalized by RGCs in both *in vitro* and *in vivo* models without the need for transfection reagents. In a rat optic nerve injury model, SC-EVs mitigated RGC degeneration, prevented RGC populations, and maintained ganglion cell complex thickness. Mechanistically, SC-EVs activated the cAMP-response element binding protein signaling pathway, crucial for regulating reactive gliosis and promoting RGC protection and axonal regeneration. These findings highlight SC-EVs as a promising cell-free therapeutic strategy and natural biomaterial for treating neurodegenerative CNS diseases [129]. Additionally, Tian *et al.* [143] enhanced EV targeting and therapeutic efficiency in cerebral ischemia by functionalizing the EV surface with Arg-Gly-Asp (RGD) peptides. Cui *et al.* [144] demonstrated that RVG-modified MSCs-EVs could alleviate memory deficits by modulating inflammatory responses in Alzheimer’s disease.

The activation of the microglial NLRP3 inflammasome plays a significant role in neuroinflammation associated with HIV-associated neurocognitive disorders. Microglia-derived EVs (MDEVs) can affect neuronal function by delivering neurotoxic mediators to recipient cells, yet the role of microglial NLRP3 in neuronal synaptic dendritic injury remains unexplored. As shown in Figure 7d, the study investigated the impact of HIV-1 Tat-induced microglial NLRP3 on neuronal synaptic dendritic injury [138].HIV-1 Tat stimulated the expression of microglial NLRP3 and IL-1β, which were packaged into MDEVs and taken up by neurons. Exposure of rat primary neurons to Tat-MDEVs resulted in decreased levels of synaptic proteins PSD95, synaptophysin, and excitatory vGLUT1, alongside increased levels of inhibitory proteins Gephyrin and GAD65, indicating impaired neuronal transmission. Additionally, Tat-MDEVs led to a reduction in dendritic spine density and alterations in spine subtype distribution (mushrooms and stubblings).

#### Reproductive system

MSC-EVs have emerged as significant tools in reproductive medicine. Recent studies have explored the use of hydrogel-encapsulated EVs to enhance the endometrial environment, address uterine adhesions, support vaginal re-epithelialization, and manage male erectile dysfunction [145]. A thin endometrial lining, a major factor in embryo implantation failure, often results in prolonged infertility and poor reproductive outcomes. Although hormone treatments have improved fertility for some women, results remain suboptimal due to damage and infection within the complex endometrial microenvironment.

**Figure 8:**
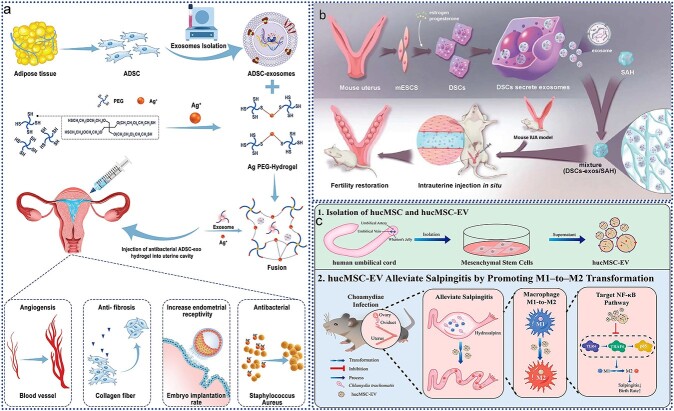
Application of EVs in the reproductive system. (**a**) schematic overview of the development of an ADSC- exos hydrogel for endometrial regeneration; adapted with permission from ref. [145], copyright 2021, Wiley-VCH; (**b**) schematic illustration of a DSC-exosome-functionalized SAH porous scaffold (DSC- exos/SAH); adapted with permission from ref. [146], copyright 2024, Wiley-VCH; (**c**) EVs derived from human umbilical cord MSCs alleviate salpingitis by promoting the conversion of M1 macrophages to M2 macrophages; adapted with permission from ref. [147], copyright 2023, the authors. MSCs mesenchymal stem cells, *hucMSC* human umbilical cord MSC, *EVs* extracellular vesicles, *ADSC* adipose-derived stem cell

As shown in Figure 8a, a study developed a multifunctional exosome hydrogel designed to protect and regenerate the endometrial microenvironment. This hydrogel was created through the dynamic coordination of silver ions (Ag+) with adipose-derived stem cell exosomes (ADSC- Exos), resulting in an injectable formulation that minimizes infection risk while preserving the antigen content and paracrine signaling capabilities of ADSC-derived cells. This innovative formulation promotes endometrial regeneration and fertility recovery through *in situ* microinjection. *In vitro*, the exosome hydrogel demonstrated significant proangiogenic effects, enhancing the proliferation and tubule formation of HUVECs by 1.87-fold and 2.2-fold, respectively. *In vivo*, the hydrogel facilitated neovascularization and tissue regeneration while mitigating local fibrosis. Importantly, the regenerated endometrial tissue showed increased receptivity for embryo implantation and led to the birth of healthy offspring. This microenvironment-protecting exosome-hydrogel system offers a practical, safe, and noninvasive approach for repairing thin endometrial linings and restoring fertility [145]. In addition, exosomes from different sources are used to treat reproductive disorders. For example, exosomes derived from mesenchymal cells encapsulated in sodium alginate hydrogel are equally capable of promoting angiogenesis, collagen remodelling and endometrial receptivity, thereby facilitating endometrial regeneration and fertility restoration [146]. In addition to this, human umbilical cord mesenchymal stem cell-derived extracellular vesicles (hucMSC-EVs) may be a promising tool for the treatment of infertility due to chronic salpingitis caused by Chlamydia trachomatis [147].

Intrauterine adhesions (IUAs), also known as Asherman’s syndrome, are characterized by fibrosis of the endometrium and scar tissue formation within the uterine cavity. This condition often leads to infertility and, in severe cases, recurrent miscarriages [148]. While cell therapies, particularly stem cell therapies, offer potential alternatives to surgical intervention, concerns about uncontrolled differentiation and tumorigenicity have limited their application. Exosomes, being more stable than their parent cells and exhibiting lower immunogenicity, have emerged as a promising alternative for treating IUAs. As shown in Figure 8b, a novel approach involves encapsulating exosomes derived from decidual stromal cells within a sodium alginate hydrogel (SAH) scaffold. This method aims to repair endometrial damage and restore fertility in a mouse model of IUA. Research findings indicate that *in situ* injection of DSC-Exos/SAH into the uterine cavity promotes uterine angiogenesis, initiates mesenchymal-epithelial transition, facilitates collagen fiber remodeling and dissolution, enhances endometrial regeneration, and improves endometrial receptivity, thereby aiding fertility restoration. RNA sequencing and advanced bioinformatics analyses suggest that miRNAs enriched in exosomes play a critical role in endometrial repair. This study provides new insights into how DSC-Exos/SAH promotes collagen ablation, endometrial regeneration, and fertility restoration, offering a novel therapeutic approach for IUA and advancing the field of regenerative medicine [146].

With rising cases of chronic salpingitis caused by *Chlamydia trachomatis* leading to infertility, there is an urgent need for effective treatments. Human umbilical cord mesenchymal stem cell-derived EVs (hucMSC-EVs) represent a promising cell-free approach. As shown in Figure 8c, this study evaluated the efficacy of hucMSC-EVs in alleviating *C. trachomatis*-induced infertility due to salpingitis through *in vivo* animal models. The study also explored the impact of hucMSC-EVs on macrophage polarization to understand the underlying molecular mechanisms. Compared with control treatments, hucMSC-EVs significantly reduced infertility caused by chlamydia-induced salpingitis. Mechanistic studies revealed that hucMSC-EVs promoted the polarization of macrophages from the M1 to the M2 phenotype via the NF-κB signaling pathway, thereby improving the local inflammatory environment in the fallopian tubes and inhibiting tubal inflammation. This approach offers a promising cell-free strategy for addressing infertility caused by chronic salpingitis [147].

Further research indicates that engineered EVs also hold therapeutic potential for reproductive disorders. Xin *et al.* [149] integrated apoptotic bodies from umbilical MSCs (UMSCs) with a HA hydrogel. These apoptotic bodies were found to modulate macrophage immune responses, stimulate cell proliferation, and enhance angiogenesis *in vitro*. In a mouse model of acute endometrial injury, implantation of the apoptotic body-loaded HA hydrogel led to increased endometrial thickness and gland count, reduced fibrosis, and improved endometrial receptivity and fertility. Additionally, Xu *et al.* [150] designed a light-triggered imide-crosslinked hydrogel containing EVs from human urine-derived stem cells, which promoted epithelial formation and angiogenesis in the rabbit vaginal mucosa. For erectile dysfunction, Liang *et al.* [151] created a thermosensitive PEG-polycaprolactone hydrogel embedded with polydopamine nanoparticles and injected ADSC-EVs into the corporal cavity using an *in situ* polymerization method. In animal models, this hydrogel facilitated sustained EV release, enhanced endothelial and neuronal cell healing, increased intracavitary pressure, and restored erectile function. Collectively, these studies highlight the potential of hydrogels containing MSC-EVs in advancing treatments for fertility enhancement and sexual dysfunction [152].

#### Digestive system

Tissue engineering represents a promising approach to tissue regeneration, significantly enhanced by stem cell therapies, which can boost and guide the regenerative process. A common strategy involves the secretion of cells or their byproducts into the medium, where they function as active biological agents [153]. EVs have emerged as a notable cell-free alternative to stem cell therapy, offering benefits such as reduced immunogenicity and preservation of biochemical activity upon storage [154, 155]. For example, exosomes derived from stem cells have demonstrated therapeutic effects in liver fibrosis. Li et al. showed that hucMSC-EVs alleviated both fibrous sac formation and inflammation in a carbon tetrachloride-induced mouse model of liver fibrosis.

**Figure 9:**
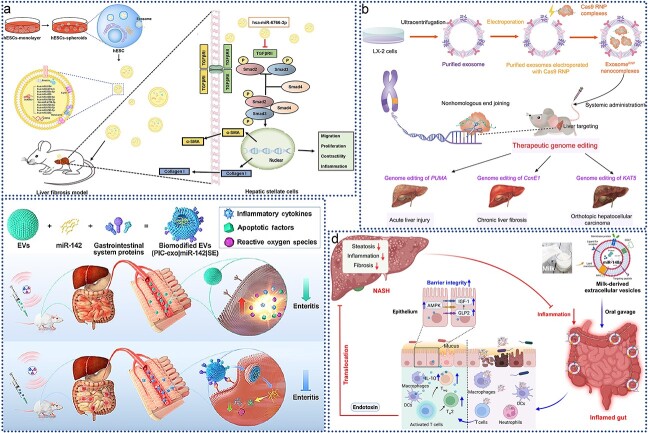
Application of EVs in the digestive system. (**a**) 3D hESC exosomes ameliorate liver fibrosis by targeting the TGFβRII-SMADS pathway; adapted with permission from ref. [156], copyright 2021, the authors; (**b**) schematic illustration of exosomes for *in vivo* delivery of Cas9 RNPs for the treatment of liver disorders; adapted with permission from ref. [157], copyright 2022, the authors; (**c**) biomodified EVs remodel the intestinal microenvironment to overcome radiation enteritis; adapted with permission from ref. [158], copyright 2023, American Chemical Society; (**d**) schematic illustration of the potential mechanisms by which milk-derived EVs (mEVs) protect gut barrier integrity in the gut-liver axis; adapted with permission from ref. [97], copyright 2023, the authors. *hESCs* human embryonic stem cells, *EVs* extracellular vesicles

Further research, as shown in Figure 9a, has explored the potential of exosomes derived from hESCs in combating fibrosis. This study compared the therapeutic efficacy of exosomes obtained from three-dimensional (3D) hESC spheroids with those from monolayer hESCs cultures. The 3D cell spheroids, which more closely mimic *in vivo* conditions, demonstrated superior regenerative potential. The results indicated that miR-6766-3p, found in exosomes from 3D hESC spheroids, inhibited TGF-βRI expression, reduced LX2 cell activation, and suppressed liver fibrosis by deactivating SMAD signaling. These findings suggest that miR-6766-3p-enriched exosomes from 3D hESCs could serve as a novel antifibrotic agent for treating chronic liver diseases. This study highlights the potential of 3D-derived exosomes as natural nanoparticles capable of delivering antifibrotic miR-6766-3p to mitigate liver injury [156].

CRISPR-Cas9 gene editing has emerged as a powerful therapeutic tool, but its clinical application is hindered by the challenge of delivering gene-editing components, especially to specific tissues. While Cas9 ribonucleoproteins (RNPs) offer significant advantages, their large size poses a challenge for current delivery vectors. As shown in Figure 9b, a novel delivery system called exosome RNPs has been developed, where Cas9 RNPs are encapsulated within exosomes isolated from hepatic stellate cells through electroporation. *In vitro* studies demonstrated that exosome RNPs effectively deliver RNPs into the cytoplasm, while *in vivo* experiments showed specific accumulation in liver tissue. Targeting genes like p53 upregulated modulator of apoptosis (PUMA), cyclin E1, and K(lysine) acetyltransferase 5 (KAT5) in mouse models of acute liver injury, chronic liver fibrosis, and HCC, exosome RNPs exhibited significant therapeutic potential. This system offers a promising platform for precise and tissue-specific gene therapy for liver diseases [157].

Circulating extracellular EVs have been implicated in shaping tissue microenvironments. Li et al. explored radiation protection strategies using small EVs (exosomes) in the context of radiation-induced intestinal injury. As shown in Figure 9c, exosomes from donor mice exposed to total body irradiation (TBI) provided protection against TBI-induced mortality and reduced gastrointestinal toxicity. Further analysis identified miRNA-142-5p as a key functional molecule in exosomes from TBI-exposed donor mice and radiotherapy patients. miRNA-142-5p was found to protect intestinal epithelial cells from radiation-induced apoptosis and mediated protection against radiation-induced colitis by enhancing the intestinal microenvironment. The study highlights the potential of biologically modified EVs with increased miR-142 expression for improving radiation-induced colitis protection [158]. The colon plays a crucial role in the digestive system, and recent studies have highlighted the protective effects of microvesicles derived from BMSCs in a rat model of colitis induced by 2,4,6-trinitrobenzene sulfonic acid [159]. These microvesicles were shown to reduce inflammatory responses, oxidative stress, and apoptosis in a dose-dependent manner [159].

mEVs are emerging as potential nanomedicines for treating intestinal diseases, though their impact on intestinal barrier integrity has been less explored. As shown in Figure 9d, mEVs from both colostrum (CM) and human milk (HM) demonstrated similar protective effects on epithelial tight junctions *in vitro*, withstanding harsh gastrointestinal conditions, and successfully reaching the colon *in vivo*. Oral administration of mEVs was found to restore intestinal barrier integrity at multiple levels—mucosal, epithelial, and immune barriers—thereby preventing endotoxin translocation to the liver in models of chemically induced colitis and diet-induced nonalcoholic steatohepatitis. These findings suggest that oral mEVs could be a promising approach for treating intestinal inflammation and related metabolic diseases by maintaining intestinal barrier integrity [97].

Periodontitis, a chronic infectious disease characterized by alveolar bone loss and progressive resorption, presents a significant clinical challenge, especially in severe cases. While achieving complete recovery of periodontal tissue structure and function is difficult, MSC-EVs have shown potential in promoting periodontal regeneration. Liu et al. demonstrated that BMSC-EVs may influence the OPG-RANKL signaling pathway, which is crucial for regulating osteoclast function, macrophage polarization, and the inflammatory immune response. Hydrogels incorporating BMSC-EVs have been shown to significantly enhance periodontal tissue regeneration [160]. Shen *et al.* [161] used chitosan hydrogels loaded with dental pulp stem cell-derived exosomes (DPSC-Exos) to treat periodontitis, showing that DPSC-Exos can modulate macrophage phenotypes and accelerate the healing of alveolar bone and periodontal epithelium while inhibiting disease progression. Additionally, Shi et al. reported that pre-treating DPSC-Exos with lipopolysaccharide further promoted periodontal regeneration. These findings underscore the therapeutic potential of MSC-EVs in periodontitis, highlighting their ability to modulate immune responses and stimulate tissue repair, thus offering new possibilities for regenerative dentistry [162].

In summary, EVs hold significant potential for various applications in the digestive system, warranting further research and exploration.

### Clinical translation and challenges

EVs have shown significant promise in regenerative medicine, with evidence suggesting they can match or even surpass the efficacy of their parent cells in promoting tissue regeneration [163]. However, translating EVs into approved clinical treatments faces several challenges. One major obstacle is the large-scale production of homogeneous, pure, and specific EV subtypes. EVs encompass a broad range of vesicles, varying in size from 30 to 1000 nm, with substantial overlap in size distributions across different vesicle types. Currently, there is no universally accepted standard for classifying these vesicles, and methods for their separation remain contentious, complicating the development of standardized EV-based therapies. To address the heterogeneity of EVs and prepare clinical-grade products, there is an urgent need for standardization and industrialization of EVs preparation and analysis techniques. Variations in cell culture conditions, such as changes in oxygen concentration or pH, can affect EVs composition and stability. Therefore, developing standardized production, purification, and quality control technologies is crucial for stabilizing EVs function and facilitating their clinical application. Efforts to scale up production include using battery factories for mass production and exploring bionic vesicles, which mimic EVs structures and functions using nanovesicles with uniform sizes. These advancements could overcome the limitations of natural EVs and open new possibilities [34]. The field of EVs engineering is rapidly evolving, with various strategies such as incubation, transfection, electroporation, ultrasound, and *in situ* synthesis being explored to load specific therapeutic payloads into EVs. Despite the potential, challenges remain, including a need for a deeper understanding of the molecular mechanisms involved in therapeutic agent packaging and targeting specific lesions. Differences in loading efficiency and specificity among EV subpopulations necessitate the development of effective engineering strategies and standardized production protocols.

Understanding the precise mechanisms by which EVs mediate tissue regeneration is still a work in progress. Recent research has focused on the roles of miRNAs, proteins, and lipids within EVs, with noncoding RNAs also emerging as significant contributors to EV function. Gaining insight into how miRNAs and other molecules are selected and incorporated into EVs is crucial for harnessing their therapeutic potential.

Isolating specific EVs subpopulations from the broad spectrum of vesicles secreted by cells is a significant challenge. This complexity involves not only diverse biogenetic pathways but also distinct molecular signatures and functions of different EVs subpopulations in tissue regeneration. Understanding whether the broad efficacy of MSC-EVs is due to a universal signaling factor or a collective effect of multiple EVs subpopulations requires rigorous research, ideally at the single-particle level. Such investigations are essential to fully exploit EVs as transformative agents in regenerative medicine.

The emerging field of EV redesign focuses on incorporating desired functions into EVs. Scientists are exploring various methods, including coincubation, transfection, electroporation, ultrasound, and *in situ* synthesis, to load specific therapeutic cargoes into EVs. While these approaches hold great promise, significant challenges remain. For instance, variations in loading efficiency and specificity among different EVs populations need further investigation. The overexpression of therapeutic molecules in EVs-secreting cells can also impact the biology of these cells, potentially affecting the overall efficacy of the EVs. Understanding these molecular mechanisms is crucial for developing effective engineering strategies and standardized production protocols for EVs. Extensive reviews by other researchers have addressed issues related to EVs cargo engineering, such as the retention of small molecules within EVs, underscoring the need for ongoing research in this area.

Due to the complexity and heterogeneity of EV preparations, ensuring high-quality, clinical-grade vesicle products is crucial. Quality control encompasses several key aspects beyond standard parameters such as vesicle size, morphology, and the presence of EVs-specific protein markers like CD63, CD81, and CD9. Comprehensive evaluation includes the assessment of total protein and RNA composition through proteomic and transcriptomic analyses, which are more advanced than typical laboratory procedures. To guarantee purity and consistency across different batches, targeted molecular analyses are conducted to monitor characteristic markers specific to the parental cells from which the EVs are derived. As the field progresses, emphasis is shifting toward quantifying the molecules that drive the functional effects of EVs, which is crucial for assessing the consistency and efficacy of bulk-produced preparations. Ensuring effective and uniform product performance over time is critical. *In vitro* functional assays are employed to detect EVs titers and are typically tailored to the specific application and intended function of the EVs. For example, MSC-EV products are often evaluated for their immunomodulatory properties, as they are commonly aimed at tissue repair and regeneration through inflammation modulation. To further validate EVs formulations, establishing and routinely applying protocols that clarify the mechanisms of action underlying their functional effects is imperative. This approach is vital for advancing the clinical translation of EV-based therapies.

Additionally, RNA-based nucleic acid functionalization within EVs faces challenges, such as RNA instability, which can lead to degradation and functional failure. There are also hurdles related to modification methods, selection of appropriate EVs, and obstacles in clinical translation. Addressing these issues is crucial for the successful development and implementation of EV-based nucleic acid therapies [33].

Neurological diseases and autoimmune disorders, particularly those associated with exacerbated inflammatory processes, are key areas where EV-based therapies are being actively developed. Genetic diseases are also prime candidates for early EV treatment, especially with engineered EVs, as these naturally secreted vesicles are considered safer delivery vehicles for RNA or gene-editing tools compared with synthetic nanoparticles. Currently, several companies, including Capricor Therapeutics, Aegle Therapeutics, United Therapeutics, and Exopharm, are conducting clinical trials using EVs for tissue repair. Meanwhile, other companies like Infusio, Exocel Bio, and ExoCoBio already offer EV-based treatment options or commercially available EV products. Interestingly, the regenerative properties of EVs are also gaining attention in the cosmetics sector due to their potential applications in skin and tissue rejuvenation [164].

There are two notable publications detailing the clinical use of MSC-EVs. The first article reports on a patient with refractory graft-versus-host disease who received four units of bone marrow MSC-derived miniature EVs via intravenous injection. One unit of EVs is defined as the fraction recovered from the supernatant of 4 × 10^7^ BM-MSCs treated for 48 h, filtered through a 0.22 μm membrane, precipitated with polyethylene glycol, and ultracentrifuged at 100 000 g for 2 h. To minimize potential side effects, the patient initially received one-tenth of a unit, with the dose gradually increasing every 2–3 days until reaching a total of four units. No adverse events were observed within 14 days of administration, and there was a significant improvement in clinical symptoms, indicating both safety and potential efficacy of this EV-based treatment [165]. The second article concerns a randomized, placebo-controlled clinical trial evaluating the safety and efficacy of umbilical cord-derived MSC-EVs (UC-MSC-EVs) in 40 patients with stage III and IV chronic kidney disease. The UC-MSC-conditioned supernatant was collected at 100 000 g using two UC steps for 1 h each. Patients received two injections of 100 μg EVs/kg body weight, 1 week apart, with the first injection administered intravenously and the second via the intrarenal artery. No adverse events were recorded, and during the 12-month follow-up, there was a significant improvement in overall kidney function. Additionally, levels of TGFβ1 and IL10 were significantly elevated alongside clinical improvement, suggesting an immunomodulatory effect. While these early clinical results are promising, it is important to note that most of the ongoing clinical trials are Phase 1 studies focused on the feasibility and safety of EV administration across different conditions. To date, only one Phase 2/3 trial has been completed, demonstrating the safety (primary endpoint) and efficacy (secondary endpoint) of UC-MSC-EVs in chronic kidney disease patients, evidenced by a 50% reduction in serum creatinine levels and a two-fold increase in estimated glomerular filtration rate [166]. To definitively establish the therapeutic value of MSC-EVs, additional randomized, double-blind Phase 2 and 3 clinical trials are necessary.

### Prospects

The industrialization and commercialization of engineered EVs face several challenges, including production costs, quality control, and market acceptance. Bacterial EVs (BEVs), which are easily obtained through bacterial fermentation, offer a promising avenue due to their scalability, low cost, and environmental friendliness, making the industrial production of pure EVs feasible [167, 168]. Additionally, synthetic biology can be leveraged to enhance bacteria and their BEVs with new functionalities. Clinical trials are already exploring the potential of biotherapeutic bacteria, including human symbiotic bacteria such as *Escherichia coli* Nissle 1917 [169], Myxococcus, and *Lactobacillus rhamnosus*. Pure EVs derived from probiotics hold significant promise in biomedical applications, particularly due to their safety profile, as they do not contain live bacteria. Both oral and intravenous administration of these EVs have shown good tolerability and minimal immunogenic reactions [170–172]. Consequently, there is increasing focus on “EVs from nonmammalian cells, especially pure EVs”, within the scientific community. The industrialization and marketing of engineered EVs for regenerative medicine are promising areas of development, with technology maturing and the market expanding.

However, the application of EEVs in regenerative medicine brings forth ethical and regulatory challenges that must not be overlooked. The safety and efficacy of engineered EVs as novel therapeutics are primary ethical concerns. Ensuring the stability, targeting accuracy, and absence of side effects of EVs *in vivo* remains a significant challenge for researchers and medical institutions. The development and use of engineered EVs as new biological products require a complex regulatory process. Simplifying this approval process while ensuring product safety and efficacy is a major regulatory challenge. Moving forward, it is crucial to establish robust ethical review mechanisms to strictly oversee clinical trials and treatments involving engineered EVs. Enhancing the ethical literacy of researchers and healthcare professionals through education and training is also vital. Additionally, the regulatory framework should be continuously updated to keep pace with advancements in engineered EV technology and market demands. Clear delineation of regulatory responsibilities and authority is necessary to ensure that regulations remain relevant and actionable.

In future research, it is imperative to establish a robust ethical review mechanism to rigorously oversee the clinical trials and treatment processes involving engineered EVs. Enhancing the ethical standards of researchers and medical professionals through comprehensive ethics education and training is equally important. The regulatory framework should be continuously revised and improved in response to advancements in engineered EV technology and evolving market demands. Clear delineation of regulatory responsibilities and authority is essential to ensure that regulations are both relevant and operable. Ethically, researchers must prioritize informed consent and privacy protection for patients. In clinical trials, it is crucial to ensure that patients fully understand the potential risks and benefits of participation and voluntarily provide informed consent. Safeguarding patients’ personal information and privacy is also vital, necessitating the implementation of stringent data management and protection measures. From a regulatory perspective, the production process of engineered EVs must adhere to rigorous quality control standards. Regulators should develop comprehensive guidelines and standards that address the preparation, purification, characterization, and storage of EVs. Additionally, an effective evaluation system should be established to scientifically assess the safety and efficacy of engineered EVs, ensuring their reliability in clinical applications. By building a strong ethical and regulatory framework, enhancing the training of researchers and medical professionals, and ensuring the safety and effectiveness of engineered EVs, the widespread application of these vesicles in regenerative medicine can be advanced for the greater well-being of patients.

The use of EEVs in tissue repair and regeneration holds significant promise. The inherent ability of EEVs to facilitate intercellular communication, modulate the biological environment, and naturally integrate into biological fluids positions them as key players in cell signaling and disease modeling. The unique properties of EEVs, such as their targeted delivery capabilities, controllable cargo loading, biocompatibility, and low immunogenicity, open new avenues for tissue repair, regeneration, disease modeling, drug screening, and personalized precision medicine. However, challenges remain, particularly regarding the purification and safety assessment of EVs. Future research will likely focus on refining targeting mechanisms, improving safety profiles, and increasing production efficiency of EEVs. These efforts are expected to yield more potent therapeutic strategies to address a wide range of diseases. As the field evolves, there is strong reason to believe that EEVs will play an increasingly prominent role in medical science, particularly in the areas of tissue repair and regenerative medicine.

## Conclusions

In summary, significant progress has been made in understanding the role of EVs and EEVs in tissue remodeling and repair. The potential of EVs in both endogenous tissue remodeling and therapeutic tissue repair is increasingly recognized, leading to a rise in early clinical trials exploring EV-based therapies. Current studies have demonstrated that stem cell-derived EVs are safe, exhibit anti-inflammatory effects, and effectively treat various inflammatory conditions while promoting wound healing. However, to fully translate EVs into safe and effective cell-free therapies, a deeper understanding of the specific proteins and other molecular components within EVs is essential. This includes investigating whether their effects are subtype-specific, tissue-specific, or pleiotropic. Future research should focus on elucidating the molecular, cellular, and functional properties of different EV subtypes and related proteins in tissue repair. Such insights will be critical for identifying which protein cargoes and intracellular mechanisms are key contributors to EV-mediated tissue repair, ultimately advancing their application in regenerative medicine.

## Abbreviations

Alzheimer’s disease (AD); Asymmetric flow-field-flow fractionation (AsFlFFF); cAMP-response element binding protein (CREB); Cas9 ribonucleoproteins (RNPs); central nervous system (CNS); cyclic RGD (cRGD); dendritic cells (DCs); Density gradient centrifugation (DGC); endothelial progenitor cells (EPCs); extracellular matrix (ECM); hepatocellular carcinoma (HCC); human umbilical vein endothelial cells (HUVECs); Intrauterine adhesions (IUAs); myocardial ischemia/reperfusion (MI/R); natural killer (NK); neural stem cells (NSCs); nonalcoholic steatohepatitis (NASH); p53 upregulated modulator of apoptosis (PUMA); PEG-polycaprolactone (PCL); phosphate buffer solution (PBS); platelet-activating protein (TSP); retinal ganglion cells (RGCs); sodium alginate hydrogel (SAH); spinal cord injury (SCI); stem cell recruiting factor (SDF-1); Tangential flow filtration (TFF); total body irradiation (TBI); tricalcium phosphate (B-TCP); tumor necrosis factor receptor-associated factor 3 (Traf3); Yes1-associated transcriptional regulator (YAP1); Jun N-terminal kinase (JNK); nuclear factor κ light chain enhancer (NF-κB); K(lysine) acetyltransferase 5 (KAT5); Mesenchymal stem cells (MSCs); bone marrow MSCs (BM-MSCs); gingival MSCs (GMSCs); hair follicle MSCs (HF-MSC); human BMSCs (hBMSCs); placental MSCs (P-MSC); human induced pluripotent stem cell-derived MSCs (hiPSC-MSCs); Human umbilical cord MSC (hucMSC); umbilical MSCs (UMSCs); high-density lipoproteins (HDL); low-density lipoproteins (LDL); intermediate-density lipoproteins (IDL); very-low-density lipoproteins (VLDL); induced pluripotent stem cell (iPSC); human- iPSC (hiPSC); hyaluronic acid (HA); three-dimensional (3D); vascular endothelial growth factor (VEGF); hepatocyte growth factor (HGF); basic fibroblast growth factor (bFGF); fibroblast growth factor (FGF); nerve growth factor (NGF); insulin-like growth factor 1 (IGF-1); Extracellular vesicles (EVs); microglia-derived EVs (MDEVs); EVs from cerebrospinal fluid (CSF-EVs); Pericyte-derived EVs (PC-EVs); platelet-mimicking EVs (P-EVs); milk-derived EVs (mEVs); small EVs (sEVs); urogenic stem cell exosomes (USC-Exos); DA7R-SDF-1-EV (dual-EV); Bacterial EVs (BEVs); Schwann cell-derived EVs (SC-EVs); adipose-derived stem cell exosomes (ADSC-Exos); adipose-derived stem cell EVs (ADSC-EVs); royal jelly derived extracellular vesicles (RJ-EVs); poly(D,L-lactide-co-glycolide) (PLGA); poly(glycolic acid) (PGA); poly(lactic acid) (PLA); polycaprolactone (PCL); polyethylene glycol (PEG); polypyrrole (PPy); methacrylic anhydride-modified silk gel (SerMA); messenger RNAs (mRNAs); adipose-derived stem cells (ADSCs); hair follicle stem cells (HFSCs); dental pulp stem cell-derived exosomes (DPSCs-Exo); DSC-exosome-functionalized SAH porous scaffold (DSC-Exos/SAH); human embryonic stem cells (hESCs); Tβ4 exosomes (Tβ4-ASCs).

## References

[1] Cui Y , Zhang W, Shan J, He J, Niu Q, Zhu C. et al. Copper nanodots-based hybrid hydrogels with multiple enzyme activities for acute and infected wound repair. *Adv Healthc Mater* 2023;13:2302566. 10.1002/adhm.202302566. 37931140

[2] Whitaker R , Hernaez-Estrada B, Hernandez RM, Santos-Vizcaino E, Spiller KL Immunomodulatory biomaterials for tissue repair. *Chem Rev* 2021;121:11305–35. 10.1021/acs.chemrev.0c00895. 34415742

[3] Driskill JH , Pan D. Control of stem cell renewal and fate by YAP and TAZ. *Nat Rev Mol Cell Biol* 2023;24:895–911. 10.1038/s41580-023-00644-5. 37626124

[4] He L , Nguyen NB, Ardehali R, Zhou B Heart regeneration by endogenous stem cells and cardiomyocyte proliferation. *Circulation* 2020;142:275–91. 10.1161/CIRCULATIONAHA.119.045566. 32687441 PMC7374760

[5] Tsiapalis D , O'Driscoll L. Mesenchymal stem cell derived extracellular vesicles for tissue engineering and regenerative medicine applications. *Cells* 2020;9. 10.3390/cells9040991 PMC7226943 32316248

[6] Zhao X , Zhang W, Fan J, Chen X, Wang X Application of mesenchymal stem cell exosomes in the treatment of skin wounds. *Smart Mater Med* 2023;4:578–89. 10.1016/j.smaim.2023.04.006.

[7] Wang Y , Fang J, Liu B, Shao C, Shi Y Reciprocal regulation of mesenchymal stem cells and immune responses. *Cell Stem Cell* 2022;29:1515–30. 10.1016/j.stem.2022.10.001. 36332569

[8] Pischiutta F , Caruso E, Lugo A, Cavaleiro H, Stocchetti N, Citerio G. et al. Systematic review and meta-analysis of preclinical studies testing mesenchymal stromal cells for traumatic brain injury. *NPJ Regen Med* 2021;6:71. 10.1038/s41536-021-00182-8. 34716332 PMC8556393

[9] Rodríguez-Pallares J , García-Garrote M, Parga JA, Labandeira-García JL Combined cell-based therapy strategies for the treatment of Parkinson's disease: focus on mesenchymal stromal cells. *Neural Regen Res* 2023;18:478–84. 10.4103/1673-5374.350193. 36018150 PMC9727452

[10] Pang QM , Deng KQ, Zhang M, Wu XC, Yang RL, Fu SP. et al. Multiple strategies enhance the efficacy of MSCs transplantation for spinal cord injury. *Biomed Pharmacother* 2023;157:114011. 10.1016/j.biopha.2022.114011. 36410123

[11] Li A , Guo F, Pan Q, Chen S, Chen J, Liu HF. et al. Mesenchymal stem cell therapy: hope for patients with systemic lupus erythematosus. *Front Immunol* 2021;12:728190. 10.3389/fimmu.2021.728190. 34659214 PMC8516390

[12] Luchetti F , Carloni S, Nasoni MG, Reiter RJ, Balduini W Melatonin, tunneling nanotubes, mesenchymal cells, and tissue regeneration. *Neural Regen Res* 2023;18:760–2. 10.4103/1673-5374.353480. 36204833 PMC9700085

[13] Caplan H , Olson SD, Kumar A, George M, Prabhakara KS, Wenzel P. et al. Mesenchymal stromal cell therapeutic delivery: translational challenges to clinical application. *Front Immunol* 2019;10:1645. 10.3389/fimmu.2019.01645. 31417542 PMC6685059

[14] Zhou T , Yuan Z, Weng J, Pei D, du X, He C. et al. Challenges and advances in clinical applications of mesenchymal stromal cells. *J Hematol Oncol* 2021;14:24. 10.1186/s13045-021-01037-x. 33579329 PMC7880217

[15] O'Brien TJ , Hollinshead F, Goodrich LR. Extracellular vesicles in the treatment and prevention of osteoarthritis: can horses help us translate this therapy to humans? *Extracell Vesicles Circ Nucl Acids.* 2023;4:151–69. 10.20517/evcna.2023.11. 37829144 PMC10568983

[16] Roefs MT , Sluijter JPG, Vader P. Extracellular vesicle-associated proteins in tissue repair. *Trends Cell Biol* 2020;30:990–1013. 10.1016/j.tcb.2020.09.009. 33069512

[17] Malda J , Boere J, van de Lest CHA, van Weeren PR, Wauben MHM Extracellular vesicles—new tool for joint repair and regeneration. *Nat Rev Rheumatol* 2016;12:243–9. 10.1038/nrrheum.2015.170. 26729461 PMC7116208

[18] Zhang K , Li R, Chen X, Yan H, Li H, Zhao X. et al. Renal endothelial cell-targeted extracellular vesicles protect the kidney from ischemic injury. *Adv Sci (Weinh)* 2023;10:e2204626. 10.1002/advs.202204626. 36416304 PMC9875634

[19] Brennan M , Layrolle P, Mooney DJ. Biomaterials functionalized with MSC secreted extracellular vesicles and soluble factors for tissue regeneration. *Adv Funct Mater* 2020;30:1909125. 10.1002/adfm.201909125. PMC7494127 32952493

[20] Tan D , Zhu W, Liu L, Pan Y, Xu Y, Huang Q. et al. In situ formed scaffold with royal jelly-derived extracellular vesicles for wound healing. *Theranostics* 2023;13:2811–24. 10.7150/thno.84665. 37284440 PMC10240823

[21] He X , Wang Y, Liu Z, Weng Y, Chen S, Pan Q. et al. Osteoporosis treatment using stem cell-derived exosomes: a systematic review and meta-analysis of preclinical studies. *Stem Cell Res Ther* 2023;14:72. 10.1186/s13287-023-03317-4. 37038180 PMC10088147

[22] Zhou H , Qi YX, Zhu CH, Li A, Pei DD Mesenchymal stem cell-derived extracellular vesicles for treatment of bone loss within periodontitis in pre-clinical animal models: a meta-analysis. *BMC Oral Health* 2023;23:701. 10.1186/s12903-023-03398-w. 37773120 PMC10540343

[23] Chu M , Wang H, Bian L. et al. Nebulization therapy with umbilical cord mesenchymal stem cell-derived exosomes for COVID-19 pneumonia. *Stem Cell Rev Rep* 2022;18:2152–63. 10.1007/s12015-022-10398-w. 35665467 PMC9166932

[24] Li P , Yin R, Chen Y, Chang J, Yang L, Liu X. et al. Engineered extracellular vesicles for ischemic stroke: a systematic review and meta-analysis of preclinical studies. *J Nanobiotechnology* 2023;21:396. 10.1186/s12951-023-02114-8. 37904204 PMC10617166

[25] Kou M , Huang L, Yang J, Chiang Z, Chen S, Liu J. et al. Mesenchymal stem cell-derived extracellular vesicles for immunomodulation and regeneration: a next generation therapeutic tool? *Cell Death Dis* 2022;13:580. 10.1038/s41419-022-05034-x. 35787632 PMC9252569

[26] Hu Z , Wang W, Lin Y, Guo H, Chen Y, Wang J. et al. Extracellular vesicle-inspired therapeutic strategies for the COVID-19. *Adv Healthc Mater.* 2024;e2402103. 10.1002/adhm.202402103. 38923772

[27] Raposo G , Stahl PD. Extracellular vesicles—on the cusp of a new language in the biological sciences. *Extracell Vesicles Circ Nucl Acids.* 2023;4:240–54. 10.20517/evcna.2023.18. 38288044 PMC10824536

[28] Pan Z , Sun W, Chen Y, Tang H, Lin W, Chen J. et al. Extracellular vesicles in tissue engineering: biology and engineered strategy. *Adv Healthc Mater* 2022;11:e2201384. 10.1002/adhm.202201384. 36053562

[29] Alvarez-Erviti L , Seow Y, Yin H, Betts C, Lakhal S, Wood MJA Delivery of siRNA to the mouse brain by systemic injection of targeted exosomes. *Nat Biotechnol* 2011;29:341–5. 10.1038/nbt.1807. 21423189

[30] Herrmann IK , Wood MJA, Fuhrmann G. Extracellular vesicles as a next-generation drug delivery platform. *Nat Nanotechnol* 2021;16:748–59. 10.1038/s41565-021-00931-2. 34211166

[31] Ding J-Y , Chen M-J, Wu L-F, Shu GF, Fang SJ, Li ZY. et al. Mesenchymal stem cell-derived extracellular vesicles in skin wound healing: roles, opportunities and challenges. *Mil Med Res* 2023;10:36. 10.1186/s40779-023-00472-w. 37587531 PMC10433599

[32] Gupta D , Wiklander OPB, Wood MJA, el-Andaloussi S Biodistribution of therapeutic extracellular vesicles. *Extracell Vesicles Circ Nucl Acids.* 2023;4:170–90. 10.20517/evcna.2023.12. PMC11648525 39697988

[33] Liu C , He D, Cen H, Chen H, Li L, Nie G. et al. Nucleic acid functionalized extracellular vesicles as promising therapeutic systems for nanomedicine. *Extracell Vesicles Circ Nucl Acids.* 2022;3:14–30. 10.20517/evcna.2021.21. PMC11648500 39697871

[34] Liu C , Wang Y, Li L, He D, Chi J, Li Q. et al. Engineered extracellular vesicles and their mimetics for cancer immunotherapy. *J Control Release* 2022;349:679–98. 10.1016/j.jconrel.2022.05.062. 35878728

[35] Liu C , He D, Li L, Zhang S, Wang L, Fan Z. et al. Extracellular vesicles in pancreatic cancer immune escape: emerging roles and mechanisms. *Pharmacol Res* 2022;183:106364. 10.1016/j.phrs.2022.106364. 35901939

[36] Silachev DN , Goryunov KV, Shpilyuk MA, Beznoschenko OS, Morozova NY, Kraevaya EE. et al. Effect of MSCs and MSC-derived extracellular vesicles on human blood coagulation. *Cells* 2019;8:8. 10.3390/cells8030258. PMC6468445 30893822

[37] Su J, Wei Q, Ma K, Wang Y, Hu W, Meng H. et al. P-MSC-derived extracellular vesicles facilitate diabetic wound healing via miR-145-5p/CDKN1A-mediated functional improvements of high glucose-induced senescent fibroblasts. Burns Trauma. 2023;11:tkad010. 10.1093/burnst/tkad010. PMC10583213 37860579

[38] Han X , Wu P, Li L, Sahal HM, Ji C, Zhang J. et al. Exosomes derived from autologous dermal fibroblasts promote diabetic cutaneous wound healing through the Akt/β-catenin pathway. *Cell Cycle* 2021;20:616–29. 10.1080/15384101.2021.1894813. 33685347 PMC8018430

[39] Wu P , Zhang B, Shi H, Qian H, Xu W MSC-exosome: a novel cell-free therapy for cutaneous regeneration. *Cytotherapy* 2018;20:291–301. 10.1016/j.jcyt.2017.11.002. 29434006

[40] Wang M, Wu P, Huang J, Liu W, Qian H, Sun Y. et al. Skin cell-derived extracellular vesicles: a promising therapeutic strategy for cutaneous injury. Burns Trauma. 2022;10:tkac037. 10.1093/burnst/tkac037. PMC9580071 36267497

[41] Rajan TS , Diomede F, Bramanti P, Trubiani O, Mazzon E Conditioned medium from human gingival mesenchymal stem cells protects motor-neuron-like NSC-34 cells against scratch-injury-induced cell death. *Int J Immunopathol Pharmacol* 2017;30:383–94. 10.1177/0394632017740976. 29140156 PMC5806806

[42] Cho BS, Kim JO, Ha DH, Yi YW. Exosomes derived from human adipose tissue-derived mesenchymal stem cells alleviate atopic dermatitis. Stem Cell Res Ther. 2018;9:187. 10.1186/s13287-018-0939-5. 10.1186/s13287-018-0939-5 PMC6042362 29996938

[43] Haumann M , Stripp ST. The molecular proceedings of biological hydrogen turnover. *Acc Chem Res* 2018;51:1755–63. 10.1021/acs.accounts.8b00109. 30001117

[44] Yoo AS , Sun AX, Li L, Shcheglovitov A, Portmann T, Li Y. et al. MicroRNA-mediated conversion of human fibroblasts to neurons. *Nature* 2011;476:228–31. 10.1038/nature10323. 21753754 PMC3348862

[45] Mao G , Zhang Z, Hu S, Zhang Z, Chang Z, Huang Z. et al. Exosomes derived from miR-92a-3p-overexpressing human mesenchymal stem cells enhance chondrogenesis and suppress cartilage degradation via targeting WNT5A. *Stem Cell Res Ther* 2018;9:247. 10.1186/s13287-018-1004-0. 30257711 PMC6158854

[46] Munir J , Yoon JK, Ryu S. Therapeutic miRNA-enriched extracellular vesicles: current approaches and future prospects. *Cells* 2020;9:2271. 10.3390/cells9102271. PMC7601381 33050562

[47] Aliotta JM , Pereira M, Wen S, Dooner MS, Del Tatto M, Papa E. et al. Exosomes induce and reverse monocrotaline-induced pulmonary hypertension in mice. *Cardiovasc Res* 2016;110:319–30. 10.1093/cvr/cvw054. 26980205 PMC4872877

[48] Maggio S , Ceccaroli P, Polidori E, Cioccoloni A, Stocchi V Signal exchange through extracellular vesicles in neuromuscular junction establishment and maintenance: from physiology to pathology. *Int J Mol Sci* 2019;20:20. 10.3390/ijms20112804. PMC6600513 31181747

[49] Valadi H , Ekström K, Bossios A, Sjöstrand M, Lee JJ, Lötvall JO Exosome-mediated transfer of mRNAs and microRNAs is a novel mechanism of genetic exchange between cells. *Nat Cell Biol* 2007;9:654–9. 10.1038/ncb1596. 17486113

[50] Figliolini F , Ranghino A, Grange C, Cedrino M, Tapparo M, Cavallari C. et al. Extracellular vesicles from adipose stem cells prevent muscle damage and inflammation in a mouse model of hind limb ischemia. *rterioscler Thromb Vasc Biol* 2020;40:239–54. 10.1161/ATVBAHA.119.313506. 31665908

[51] Borges FT , Melo SA, Özdemir BC, Kato N, Revuelta I, Miller CA. et al. TGF-β1–containing exosomes from injured epithelial cells activate fibroblasts to initiate tissue regenerative responses and fibrosis. *J Am Soc Nephrol* 2013;24:385–92. 10.1681/ASN.2012101031. 23274427 PMC3582210

[52] Kim HS , Choi DY, Yun SJ, Choi SM, Kang JW, Jung JW. et al. Proteomic analysis of microvesicles derived from human mesenchymal stem cells. *J Proteome Res* 2012;11:839–49. 10.1021/pr200682z. 22148876

[53] Qiu G , Zheng G, Ge M, Wang J, Huang R, Shu Q. et al. Functional proteins of mesenchymal stem cell-derived extracellular vesicles. *Stem Cell Res Ther* 2019;10:359. 10.1186/s13287-019-1484-6. 31779700 PMC6883709

[54] Skotland T , Sandvig K, Llorente A. Lipids in exosomes: current knowledge and the way forward. *Prog Lipid Res* 2017;66:30–41. 10.1016/j.plipres.2017.03.001. 28342835

[55] Hu Q , Su H, Li J, Lyon C, Tang W, Wan M. et al. Clinical applications of exosome membrane proteins. *Precis Clin Med* 2020;3:54–66. 10.1093/pcmedi/pbaa007. 32257533 PMC7099650

[56] Battistelli M , Falcieri E. Apoptotic bodies: particular extracellular vesicles involved in intercellular communication. *Behav Biol* 2020;9:14. 10.3390/biology9010021. PMC7168913 31968627

[57] Lai JJ , Chau ZL, Chen SY, Hill JJ, Korpany KV, Liang NW. et al. Exosome processing and characterization approaches for research and technology development. *Adv Sci (Weinh).* 2022;9:e2103222. 10.1002/advs.202103222. 35332686 PMC9130923

[58] Mathieu M , Martin-Jaular L, Lavieu G, Théry C Specificities of secretion and uptake of exosomes and other extracellular vesicles for cell-to-cell communication. *Nat Cell Biol* 2019;21:9–17. 10.1038/s41556-018-0250-9. 30602770

[59] Onódi Z , Pelyhe C, Terézia Nagy C, Brenner GB, Almási L, Kittel Á. et al. Isolation of high-purity extracellular vesicles by the combination of iodixanol density gradient ultracentrifugation and bind-elute chromatography from blood plasma. *Front Physiol* 2018;9:1479. 10.3389/fphys.2018.01479. 30405435 PMC6206048

[60] Saludes JP , Morton LA, Coulup SK, Fiorini Z, Cook BM, Beninson L. et al. Multivalency amplifies the selection and affinity of bradykinin-derived peptides for lipid nanovesicles. *Mol BioSyst* 2013;9:2005–9. 10.1039/c3mb70109c. 23715428 PMC3764994

[61] McNamara RP , Caro-Vegas CP, Costantini LM, Landis JT, Griffith JD, Damania BA. et al. Large-scale, cross-flow based isolation of highly pure and endocytosis-competent extracellular vesicles. *J Extracell Vesicles* 2018;7:1541396. 10.1080/20013078.2018.1541396. 30533204 PMC6282418

[62] Busatto S , Vilanilam G, Ticer T, Lin WL, Dickson DW, Shapiro S. et al. Tangential flow filtration for highly efficient concentration of extracellular vesicles from large volumes of fluid. *Cells* 2018;7:273. 10.3390/cells7120273. PMC6315734 30558352

[63] Musante L , Tataruch D, Gu D, Benito-Martin A, Calzaferri G, Aherne S. et al. A simplified method to recover urinary vesicles for clinical applications, and sample banking. *Sci Rep* 2014;4:7532. 10.1038/srep07532. 25532487 PMC4274508

[64] Ju S , Mu J, Dokland T, Zhuang X, Wang Q, Jiang H. et al. Grape exosome-like nanoparticles induce intestinal stem cells and protect mice from DSS-induced colitis. *Mol Ther* 2013;21:1345–57. 10.1038/mt.2013.64. 23752315 PMC3702113

[65] Sun B , Kitchen S, Tang N, Garza A, Jacob S, Pulliam L Engineered induced-pluripotent stem cell derived monocyte extracellular vesicles alter inflammation in HIV humanized mice. *Extracell Vesicles Circ Nucl Acids.* 2022;3:118–32. 10.20517/evcna.2022.11. 37067894 PMC10104589

[66] Huang D-F , Zhang W-J, Chen J, Jiao ZG, Wang XL, Rao DY. et al. Hepatocellular carcinoma cell-derived small extracellular vesicle-associated CD147 serves as a diagnostic marker and promotes endothelial cell angiogenesis via the PI3K/Akt pathway. *Extracell Vesicles Circ Nucl Acids.* 2023;4:532–47. 10.20517/evcna.2023.30. PMC12066417 40357132

[67] Craddock VD , Cook CM, Dhillon NK. Exploring extracellular vesicles as mediators of clinical disease and vehicles for viral therapeutics: insights from the COVID-19 pandemic. *Extracell Vesicles Circ Nucl Acids.* 2022;3:172–88. 10.20517/evcna.2022.19. 35929616 PMC9348627

[68] Delcayre A , Estelles A, Sperinde J, Roulon T, Paz P, Aguilar B. et al. Exosome display technology: applications to the development of new diagnostics and therapeutics. *Blood Cells Mol Dis* 2005;35:158–68. 10.1016/j.bcmd.2005.07.003. 16087368

[69] Liu Q , Li D, Pan X, Liang Y Targeted therapy using engineered extracellular vesicles: principles and strategies for membrane modification. *J Nanobiotechnology.* 2023;21:334. 10.1186/s12951-023-02081-0. 37717008 PMC10505332

[70] Lin Y , Wu J, Gu W, Huang Y, Tong Z, Huang L. et al. Exosome-liposome hybrid nanoparticles deliver CRISPR/Cas9 system in MSCs. *Adv Sci (Weinh).* 2018;5:1700611. 10.1002/advs.201700611. 29721412 PMC5908366

[71] Liang Y , Xu X, Xu L, Iqbal Z, Ouyang K, Zhang H. et al. Chondrocyte-specific genomic editing enabled by hybrid exosomes for osteoarthritis treatment. *Theranostics* 2022;12:4866–78. 10.7150/thno.69368. 35836795 PMC9274754

[72] Lv Q , Cheng L, Lu Y, Zhang X, Wang Y, Deng J. et al. Thermosensitive exosome-liposome hybrid nanoparticle-mediated Chemoimmunotherapy for improved treatment of metastatic peritoneal cancer. *Adv Sci (Weinh).* 2020;7:2000515. 10.1002/advs.202000515. 32999828 PMC7509655

[73] Chen A , Tian H, Yang N, Zhang Z, Yang GY, Cui W. et al. Towards extracellular vesicle delivery systems for tissue regeneration: material design at the molecular level. *Extracell Vesicles Circ Nucl Acids.* 2022;3:306–39. 10.20517/evcna.2022.37. PMC11648451 39697358

[74] Shi Q , Qian Z, Liu D, Sun J, Wang X, Liu H. et al. GMSC-derived exosomes combined with a chitosan/silk hydrogel sponge accelerates wound healing in a diabetic rat skin defect model. *Front Physiol* 2017;8:904. 10.3389/fphys.2017.00904. 29163228 PMC5681946

[75] Liu X, Yang Y, Li Y, Niu X, Zhao B, Wang Y. et al. Integration of stem cell-derived exosomes with in situ hydrogel glue as a promising tissue patch for articular cartilage regeneration. Nanoscale. 2017;9:4430–4438. 10.1039/c7nr00352h. 10.1039/c7nr00352h 28300264

[76] Chew JRJ , Chuah SJ, Teo KYW, Zhang S, Lai RC, Fu JH. et al. Mesenchymal stem cell exosomes enhance periodontal ligament cell functions and promote periodontal regeneration. *Acta Biomater* 2019;89:252–64. 10.1016/j.actbio.2019.03.021. 30878447

[77] Jiao K , Liu C, Basu S, Raveendran N, Nakano T, Ivanovski S. et al. Bioprinting extracellular vesicles as a "cell-free" regenerative medicine approach. *Extracell Vesicles Circ Nucl Acids* 2023;4:218–39. 10.20517/evcna.2023.19. PMC11648406 39697984

[78] Varkey M , Visscher DO, van Zuijlen PPM, Atala A, Yoo JJ Skin bioprinting: the future of burn wound reconstruction? *Burns Trauma* 2019;7:4. 10.1186/s41038-019-0142-7. 30805375 PMC6371568

[79] Chen P, Zheng L, Wang Y, Tao M, Xie Z, Xia C. et al.. Desktop-stereolithography 3D printing of a radially oriented extracellular matrix/mesenchymal stem cell exosome bioink for osteochondral defect regeneration. Theranostics. 2019;9:2439–2459. 31131046 10.7150/thno.31017 PMC6525998

[80] Mathiyalagan P , Sahoo S. Exosomes-based gene therapy for microRNA delivery. *Methods Mol Biol* 2017;1521:139–52. 10.1007/978-1-4939-6588-5_9. 27910046 PMC5502074

[81] Guo S , Perets N, Betzer O, Ben-Shaul S, Sheinin A, Michaelevski I. et al. Intranasal delivery of mesenchymal stem cell derived exosomes loaded with phosphatase and tensin homolog siRNA repairs complete spinal cord injury. *ACS Nano* 2019;13:10015–28. 10.1021/acsnano.9b01892. 31454225

[82] Tang J , Cui X, Zhang Z, Xu Y, Guo J, Soliman BG. et al. Injection-free delivery of MSC-derived extracellular vesicles for myocardial infarction therapeutics. *Adv Healthc Mater.* 2022;11:e2100312. 34310068 10.1002/adhm.202100312

[83] Zhang Y , Xie Y, Hao Z, Zhou P, Wang P, Fang S. et al. Umbilical mesenchymal stem cell-derived exosome-encapsulated hydrogels accelerate bone repair by enhancing angiogenesis. *ACS Appl Mater Interfaces* 2021;13:18472–87. 10.1021/acsami.0c22671. 33856781

[84] Gang D , Yu CJ, Zhu S, Zhu P, Nasser MI Application of mesenchymal stem cell-derived exosomes in kidney diseases. *Cell Immunol* 2021;364:104358. 10.1016/j.cellimm.2021.104358. 33839596

[85] Yang Z , Shi J, Xie J, Wang Y, Sun J, Liu T. et al. Large-scale generation of functional mRNA-encapsulating exosomes via cellular nanoporation. *Nat Biomed Eng.* 2020;4:69–83. 10.1038/s41551-019-0485-1. 31844155 PMC7080209

[86] Ramírez OJ , Alvarez S, Contreras-Kallens P, Barrera NP, Aguayo S, Schuh CMAP Type I collagen hydrogels as a delivery matrix for royal jelly derived extracellular vesicles. *Drug Deliv* 2020;27:1308–18. 10.1080/10717544.2020.1818880. 32924637 PMC7534280

[87] Zhang B , Huang J, Liu J, Lin F, Ding Z, Xu J Injectable composite hydrogel promotes osteogenesis and angiogenesis in spinal fusion by optimizing the bone marrow mesenchymal stem cell microenvironment and exosomes secretion. *Mater Sci Eng C Mater Biol Appl* 2021;123:111782. 10.1016/j.msec.2020.111782. 33812569

[88] Jiang T , Liu S, Wu Z, Li Q, Ren S, Chen J. et al. ADSC-exo@MMP-PEG smart hydrogel promotes diabetic wound healing by optimizing cellular functions and relieving oxidative stress. *Mater Today Bio* 2022;16:100365. 10.1016/j.mtbio.2022.100365. PMC9364034 35967739

[89] Cheng J , Chen Z, Liu C, Zhong M, Wang S, Sun Y. et al. Bone mesenchymal stem cell-derived exosome-loaded injectable hydrogel for minimally invasive treatment of spinal cord injury. *Nanomedicine (Lond)* 2021;16:1567–79. 10.2217/nnm-2021-0025. 34189939

[90] Mardpour S , Ghanian MH, Sadeghi-abandansari H, Mardpour S, Nazari A, Shekari F. et al. Hydrogel-mediated sustained systemic delivery of mesenchymal stem cell-derived extracellular vesicles improves hepatic regeneration in chronic liver failure. *ACS Appl Mater Interfaces* 2019;11:37421–33. 10.1021/acsami.9b10126. 31525863

[91] Song Y , You Y, Xu X, Lu J, Huang X, Zhang J. et al. Adipose-derived mesenchymal stem cell-derived exosomes biopotentiated extracellular matrix hydrogels accelerate diabetic wound healing and skin regeneration. *Adv Sci (Weinh)* 2023;10:e2304023. 37712174 10.1002/advs.202304023 PMC10602544

[92] Li C , Qin T, Jin Y, Hu J, Yuan F, Cao Y. et al. Cerebrospinal fluid-derived extracellular vesicles after spinal cord injury promote vascular regeneration via PI3K/AKT signaling pathway. *J Orthop Translat* 2023;39:124–34. 10.1016/j.jot.2023.02.001. 36909861 PMC9999163

[93] Li Q , Song Y, Wang Q, Chen J, Gao J, Tan H. et al. Engineering extracellular vesicles with platelet membranes fusion enhanced targeted therapeutic angiogenesis in a mouse model of myocardial ischemia reperfusion. *Theranostics* 2021;11:3916–31. 10.7150/thno.52496. 33664870 PMC7914364

[94] Cao Y , Xu Y, Chen C, Xie H, Lu H, Hu J Local delivery of USC-derived exosomes harboring ANGPTL3 enhances spinal cord functional recovery after injury by promoting angiogenesis. *Stem Cell Res Ther* 2021;12:20. 10.1186/s13287-020-02078-8. 33413639 PMC7791988

[95] Ma W , Zhang H, Li S, Wang Z, Wu X, Yan R. et al. A multifunctional nanoplatform based on Fenton-like and Russell reactions of Cu, Mn bimetallic ions synergistically enhanced ROS stress for improved chemodynamic therapy. *ACS Biomater Sci Eng* 2022;8:1354–66. 10.1021/acsbiomaterials.1c01605. 35230802

[96] Yang G , Chen Q, Wen D, Chen Z, Wang J, Chen G. et al. A therapeutic microneedle patch made from hair-derived keratin for promoting hair regrowth. *ACS Nano* 2019;13:4354–60. 10.1021/acsnano.8b09573. 30942567

[97] Tong L , Zhang S, Liu Q, Huang C, Hao H, Tan MS. et al. Milk-derived extracellular vesicles protect intestinal barrier integrity in the gut-liver axis. *Sci Adv* 2023;9:eade5041. 10.1126/sciadv.ade5041. 37043568 PMC10096581

[98] Lai J , Pan Q, Chen G, Liu Y, Chen C, Pan Y. et al. Triple hybrid cellular Nanovesicles promote cardiac repair after ischemic reperfusion. *ACS Nano* 2024;18:4443–55. 10.1021/acsnano.3c10784. 38193813

[99] Lu X , Xu Z, Shu F, Wang Y, Han Y, Yang X. et al. Reactive oxygen species responsive multifunctional fusion extracellular Nanovesicles: prospective treatments for acute heart transplant rejection. *Adv Mater* 2024;e2406758, 36. 38949397 10.1002/adma.202406758

[100] Koo S , Sohn HS, Kim TH, Yang S, Jang SY, Ye S. et al. Ceria-vesicle nanohybrid therapeutic for modulation of innate and adaptive immunity in a collagen-induced arthritis model. *Nat Nanotechnol* 2023;18:1502–14. 10.1038/s41565-023-01523-y. 37884660

[101] Li X , Qiao Q, Liu X, Hu Q, Yu Y, Qin X. et al. Engineered biomimetic Nanovesicles based on neutrophils for hierarchical targeting therapy of acute respiratory distress syndrome. *ACS Nano* 2024;18:1658–77. 10.1021/acsnano.3c09848. 38166370

[102] Bai Y , Chen J, Zhang S, Xu G, Mao Z, Ding Y. et al. Inflammation-responsive cell membrane-camouflaged nanoparticles against liver fibrosis via regulating endoplasmic reticulum stress and oxidative stress. *Adv Mater* 2024;36: e2310443. 38372054 10.1002/adma.202310443

[103] Zhao Y , Li Q, Niu J, Guo E, Zhao C, Zhang J. et al. Neutrophil membrane-camouflaged polyprodrug nanomedicine for inflammation suppression in ischemic stroke therapy. *Adv Mater* 2024;36:e2311803. 38519052 10.1002/adma.202311803

[104] Qin X , Zhu L, Zhong Y, Wang Y, Wu G, Qiu J. et al. Spontaneously right-side-out-orientated coupling-driven ROS-sensitive nanoparticles on cell membrane inner leaflet for efficient renovation in vascular endothelial injury. *Adv Sci (Weinh).* 2023;10:e2205093. 10.1002/advs.202205093. 36703487 PMC9951580

[105] Yang Z , Zhang Z, Li L, Jing Z, Ma Y, Lan T. et al. Bioengineered artificial extracellular vesicles presenting PD-L1 and Gal-9 ameliorate new-onset type 1 diabetes. *Diabetes* 2024;73:1325–35. 10.2337/db23-0987. 38771941

[106] Jin X , Shan J, Zhao J, Wang T, Zhang W, Yang S. et al. Bimetallic oxide Cu–Fe3O4 nanoclusters with multiple enzymatic activities for wound infection treatment and wound healing. *Acta Biomater* 2024;173:403–19. 10.1016/j.actbio.2023.10.028. 39492500

[107] Wang W , Cui Y, Wei X, Zang Y, Chen X, Cheng L. et al. CuCo2O4 nanoflowers with multiple enzyme activities for treating bacterium-infected wounds via cuproptosis-like death. *ACS Nano* 2024;18:15845–63. 10.1021/acsnano.4c02825. 38832685

[108] Atala A , Irvine DJ, Moses M, Shaunak S Wound healing versus regeneration: role of the tissue environment in regenerative medicine. *MRS Bull* 2010;35:597–606. 10.1557/mrs2010.528 PMC3826556 24241586

[109] Zhao M , Kang M, Wang J, Yang R, Zhong X, Xie Q. et al. Stem cell-derived Nanovesicles embedded in dual-layered hydrogel for programmed ROS regulation and comprehensive tissue regeneration in burn wound healing. *Adv Mater* 2024;36:e2401369. 10.1002/adma.202401369. 38822749

[110] Liu W , Yu M, Xie D, Wang L, Ye C, Zhu Q. et al. Melatonin-stimulated MSC-derived exosomes improve diabetic wound healing through regulating macrophage M1 and M2 polarization by targeting the PTEN/AKT pathway. *Stem Cell Res Ther* 2020;11:259. 10.1186/s13287-020-01756-x. 32600435 PMC7322868

[111] Hu Y , Tao R, Chen L, Xiong Y, Xue H, Hu L. et al. Exosomes derived from pioglitazone-pretreated MSCs accelerate diabetic wound healing through enhancing angiogenesis. *J Nanobiotechnology.* 2021;19:150. 10.1186/s12951-021-00894-5. 34020670 PMC8139165

[112] Ren S , Chen J, Duscher D, Liu Y, Guo G, Kang Y. et al. Microvesicles from human adipose stem cells promote wound healing by optimizing cellular functions via AKT and ERK signaling pathways. *Stem Cell Res Ther* 2019;10:47. 10.1186/s13287-019-1152-x. 30704535 PMC6357421

[113] Fang S , Xu C, Zhang Y, Xue C, Yang C, Bi H. et al. Umbilical cord-derived mesenchymal stem cell-derived exosomal microRNAs suppress Myofibroblast differentiation by inhibiting the transforming growth factor-β/SMAD2 pathway during wound healing. *Stem Cells Transl Med* 2016;5:1425–39. 10.5966/sctm.2015-0367. 27388239 PMC5031180

[114] Sheyn D , Ben-David S, Shapiro G, de Mel S, Bez M, Ornelas L. et al. Human induced pluripotent stem cells differentiate into functional mesenchymal stem cells and repair bone defects. *Stem Cells Transl Med* 2016;5:1447–60. 10.5966/sctm.2015-0311. 27400789 PMC5070500

[115] Zhang B , Shi Y, Gong A, Pan Z, Shi H, Yang H, Fu H, Yan Y, Zhang X, Wang M, Zhu W, Qian H, Xu W. HucMSC Exosome-Delivered 14-3-3ζ Orchestrates Self-Control of the Wnt Response via Modulation of YAP During Cutaneous Regeneration. Stem Cells. 2016;34:2485–2500. 27334574 10.1002/stem.2432

[116] Littman N , Abo A. Proceedings: using stem cell therapies to Reestablish osteogenic capability for bone regeneration. *Stem Cells Transl Med* 2015;4:1247–50. 10.5966/sctm.2015-0202. 26483540 PMC4622413

[117] Furuta T , Miyaki S, Ishitobi H, Ogura T, Kato Y, Kamei N. et al. Mesenchymal stem cell-derived exosomes promote fracture healing in a mouse model. *Stem Cells Transl Med* 2016;5:1620–30. 10.5966/sctm.2015-0285. 27460850 PMC5189643

[118] Forteza-Genestra MA , Antich-Rosselló M, Ráez-Meseguer C, Sangenís AT, Calvo J, Gayà A. et al. Intra-articular injection of platelet lysate-derived extracellular vesicles recovers from knee osteoarthritis in an in vivo rat model. *J Orthop Translat* 2024;45:1–9. 10.1016/j.jot.2023.10.005. 38371711 PMC10873568

[119] Uehara N , Shibusawa N, Mikami Y, Kyumoto-Nakamura Y, Sonoda S, Kato H. et al. Bone metastatic mammary tumor cell-derived extracellular vesicles inhibit osteoblast maturation via JNK signaling. *Arch Biochem Biophys* 2023;750:109821. 10.1016/j.abb.2023.109821. 37979903

[120] Cai M , Peng H, Liu M, Huang M, Zheng W, Zhang G. et al. Vascular Pericyte-derived exosomes inhibit bone resorption via Traf3. *Int J Nanomedicine* 2023; Volume 18:7065–77. 10.2147/IJN.S438229. 38046234 PMC10693246

[121] Wang X , Zou C, Hou C, Bian Z, Jiang W, Li M. et al. Extracellular vesicles from bone marrow mesenchymal stem cells alleviate osteoporosis in mice through USP7-mediated YAP1 protein stability and the Wnt/β-catenin pathway. *Biochem Pharmacol* 2023;217:115829. 10.1016/j.bcp.2023.115829. 37748664

[122] Han S , Yang H, Ni X, Deng Y, Li Z, Xing X. et al. Programmed release of vascular endothelial growth factor and exosome from injectable chitosan nanofibrous microsphere-based PLGA-PEG-PLGA hydrogel for enhanced bone regeneration. *Int J Biol Macromol* 2023;253:126721. 10.1016/j.ijbiomac.2023.126721. 37673168

[123] Pittenger MF , Mackay AM, Beck SC, Jaiswal RK, Douglas R, Mosca JD. et al. Multilineage potential of adult human mesenchymal stem cells. *Science* 1999;284:143–7. 10.1126/science.284.5411.143. 10102814

[124] Li S , Yue Y, Wang W, Han M, Wan X, Li Q. et al. Ultrasound-activated probiotics vesicles coating for titanium implant infections through bacterial cuproptosis-like death and immunoregulation. *Adv Mater* 2024;e2405953. 10.1002/adma.202405953. 39101293

[125] Qi X , Zhang J, Yuan H, Xu Z, Li Q, Niu X. et al. Exosomes secreted by human-induced pluripotent stem cell-derived mesenchymal stem cells repair critical-sized bone defects through enhanced angiogenesis and osteogenesis in osteoporotic rats. *Int J Biol Sci* 2016;12:836–49. 10.7150/ijbs.14809. 27313497 PMC4910602

[126] Zhang J , Liu X, Li H, Chen C, Hu B, Niu X. et al. Exosomes/tricalcium phosphate combination scaffolds can enhance bone regeneration by activating the PI3K/Akt signaling pathway. *Stem Cell Res Ther* 2016;7:136. 10.1186/s13287-016-0391-3. 27650895 PMC5028974

[127] Safari B , Aghazadeh M, Davaran S, Roshangar L Exosome-loaded hydrogels: a new cell-free therapeutic approach for skin regeneration. *Eur J Pharm Biopharm* 2022;171:50–9. 10.1016/j.ejpb.2021.11.002. 34793943

[128] Zhang M , Malik AB, Rehman J. Endothelial progenitor cells and vascular repair. *Curr Opin Hematol* 2014;21:224–8. 10.1097/MOH.0000000000000041. 24637956 PMC4090051

[129] Li X , Chen C, Wei L, Li Q, Niu X, Xu Y. et al. Exosomes derived from endothelial progenitor cells attenuate vascular repair and accelerate reendothelialization by enhancing endothelial function. *Cytotherapy* 2016;18:253–62. 10.1016/j.jcyt.2015.11.009. 26794715

[130] Hu GW , Li Q, Niu X, Hu B, Liu J, Zhou SM. et al. Exosomes secreted by human-induced pluripotent stem cell-derived mesenchymal stem cells attenuate limb ischemia by promoting angiogenesis in mice. *Stem Cell Res Ther* 2015;6:10. 10.1186/scrt546. 26268554 PMC4533800

[131] Zhang HC , Liu XB, Huang S, Bi XY, Wang HX, Xie LX. et al. Microvesicles derived from human umbilical cord mesenchymal stem cells stimulated by hypoxia promote angiogenesis both in vitro and in vivo. *Stem Cells Dev* 2012;21:3289–97. 10.1089/scd.2012.0095. 22839741 PMC3516422

[132] Chen P , Ning X, Li W, Pan Y, Wang L, Li H. et al. Fabrication of Tβ4-exosome-releasing artificial stem cells for myocardial infarction therapy by improving coronary collateralization. *Bioact Mater* 2022;14:416–29. 10.1016/j.bioactmat.2022.01.029. 35386821 PMC8964820

[133] Liu S , Chen X, Bao L, Liu T, Yuan P, Yang X. et al. Treatment of infarcted heart tissue via the capture and local delivery of circulating exosomes through antibody-conjugated magnetic nanoparticles. *Nat Biomed Eng* 2020;4:1063–75. 10.1038/s41551-020-00637-1. 33159193

[134] Huang CC , Kang M, Lu Y, Shirazi S, Diaz JI, Cooper LF. et al. Functionally engineered extracellular vesicles improve bone regeneration. *Acta Biomater* 2020;109:182–94. 10.1016/j.actbio.2020.04.017. 32305445 PMC8040700

[135] Casella G , Colombo F, Finardi A, Descamps H, Ill-Raga G, Spinelli A. et al. Extracellular vesicles containing IL-4 modulate Neuroinflammation in a mouse model of multiple sclerosis. *Mol Ther* 2018;26:2107–18. 10.1016/j.ymthe.2018.06.024. 30017878 PMC6127510

[136] Ruan H , Li Y, Wang C, Jiang Y, Han Y, Li Y. et al. Click chemistry extracellular vesicle/peptide/chemokine nanocarriers for treating central nervous system injuries. *Acta Pharm Sin B* 2023;13:2202–18. 10.1016/j.apsb.2022.06.007. 37250158 PMC10213615

[137] Zhu S , Chen L, Wang M, Zhang J, Chen G, Yao Y. et al. Schwann cell-derived extracellular vesicles as a potential therapy for retinal ganglion cell degeneration. *J Control Release* 2023;363:641–56. 10.1016/j.jconrel.2023.10.012. 37820984

[138] Kannan M , Singh S, Chemparathy DT, Oladapo AA, Gawande DY, Dravid SM. et al. HIV-1 tat induced microglial EVs leads to neuronal synaptodendritic injury: microglia-neuron cross-talk in NeuroHIV. *Extracell Vesicles Circ Nucl Acids* 2022;3:133–49. 10.20517/evcna.2022.14. 36812097 PMC9937449

[139] Webb RL , Kaiser EE, Scoville SL, Thompson TA, Fatima S, Pandya C. et al. Human neural stem cell extracellular vesicles improve tissue and functional recovery in the murine thromboembolic stroke model. *Transl Stroke Res* 2018;9:530–9. 10.1007/s12975-017-0599-2. 29285679 PMC6132936

[140] Fan L , Liu C, Chen X, Zheng L, Zou Y, Wen H. et al. Exosomes-loaded electroconductive hydrogel synergistically promotes tissue repair after spinal cord injury via Immunoregulation and enhancement of myelinated axon growth. *Adv Sci (Weinh).* 2022;9:e2105586. 35253394 10.1002/advs.202105586 PMC9069372

[141] Han M , Yang H, Lu X, Li Y, Liu Z, Li F. et al. Three-dimensional-cultured MSC-derived exosome-hydrogel hybrid microneedle Array patch for spinal cord repair. *Nano Lett* 2022;22:6391–401. 10.1021/acs.nanolett.2c02259. 35876503

[142] Chen Y , Lin J, Yan W. A prosperous application of hydrogels with extracellular vesicles release for traumatic brain injury. *Front Neurol* 2022;13:908468. 10.3389/fneur.2022.908468. 35720072 PMC9201053

[143] Tian T , Zhang HX, He CP, Fan S, Zhu YL, Qi C. et al. Surface functionalized exosomes as targeted drug delivery vehicles for cerebral ischemia therapy. *Biomaterials* 2018;150:137–49. 10.1016/j.biomaterials.2017.10.012. 29040874

[144] Cui GH , Guo HD, Li H, Zhai Y, Gong ZB, Wu J. et al. RVG-modified exosomes derived from mesenchymal stem cells rescue memory deficits by regulating inflammatory responses in a mouse model of Alzheimer's disease. *Immun Ageing* 2019;16:10. 10.1186/s12979-019-0150-2. 31114624 PMC6515654

[145] Lin J , Wang Z, Huang J, Tang S, Saiding Q, Zhu Q. et al. Microenvironment-protected exosome-hydrogel for facilitating endometrial regeneration, fertility restoration, and live birth of offspring. *Small* 2021;17:e2007235. 10.1002/smll.202007235. 33590681

[146] Liang Y , Shuai Q, Zhang X, Jin S, Guo Y, Yu Z. et al. Incorporation of Decidual stromal cells derived exosomes in sodium alginate hydrogel as an innovative therapeutic strategy for advancing endometrial regeneration and reinstating fertility. *Adv Healthc Mater.* 2024;13:e2303674. 10.1002/adhm.202303674. 38315148

[147] Zhang C , Liao W, Li W, Li M, Xu X, Sun H. et al. Human umbilical cord mesenchymal stem cells derived extracellular vesicles alleviate salpingitis by promoting M1-to-M2 transformation. *Front Physiol* 2023;14:1131701. 10.3389/fphys.2023.1131701. 36875046 PMC9977816

[148] Salazar CA , Isaacson K, Morris S. A comprehensive review of Asherman's syndrome: causes, symptoms and treatment options. *Curr Opin Obstet Gynecol* 2017;29:249–56. 10.1097/GCO.0000000000000378. 28582327

[149] Xin L , Wei C, Tong X, Dai Y, Huang D, Chen J. et al. In situ delivery of apoptotic bodies derived from mesenchymal stem cells via a hyaluronic acid hydrogel: a therapy for intrauterine adhesions. *Bioact Mater* 2022;12:107–19. 10.1016/j.bioactmat.2021.10.025. 35087967 PMC8777284

[150] Xu Y , Qiu Y, Lin Q, Huang C, Li J, Chen L. et al. miR-126-3p-loaded small extracellular vesicles secreted by urine-derived stem cells released from a phototriggered imine crosslink hydrogel could enhance vaginal epithelization after vaginoplasty. *Stem Cell Res Ther* 2022;13:331. 10.1186/s13287-022-03003-x. 35870968 PMC9308191

[151] Liang L , Shen Y, Dong Z, Gu X Photoacoustic image-guided corpus cavernosum intratunical injection of adipose stem cell-derived exosomes loaded polydopamine thermosensitive hydrogel for erectile dysfunction treatment. *Bioact Mater.* 2022;9:147–56. 10.1016/j.bioactmat.2021.07.024. 34820562 PMC8586570

[152] Ju Y , Hu Y, Yang P, Xie X, Fang B Extracellular vesicle-loaded hydrogels for tissue repair and regeneration. *Mater Today Bio* 2023;18:100522. 10.1016/j.mtbio.2022.100522. PMC9803958 36593913

[153] Bjørge IM , Kim SY, Mano JF, Kalionis B, Chrzanowski W Extracellular vesicles, exosomes and shedding vesicles in regenerative medicine - a new paradigm for tissue repair. *Biomater Sci* 2017;6:60–78. 10.1039/c7bm00479f. 29184934

[154] Wu Y , Deng W, Klinke DJ II. Exosomes: improved methods to characterize their morphology, RNA content, and surface protein biomarkers. *Analyst* 2015;140:6631–42. 10.1039/C5AN00688K. 26332016 PMC4986832

[155] Konala VB , Mamidi MK, Bhonde R, das AK, Pochampally R, Pal R The current landscape of the mesenchymal stromal cell secretome: a new paradigm for cell-free regeneration. *Cytotherapy* 2016;18:13–24. 10.1016/j.jcyt.2015.10.008. 26631828 PMC4924535

[156] Wang N , Li X, Zhong Z, Qiu Y, Liu S, Wu H. et al. 3D hESC exosomes enriched with miR-6766-3p ameliorates liver fibrosis by attenuating activated stellate cells through targeting the TGFβRII-SMADS pathway. *J Nanobiotechnology* 2021;19:437. 10.1186/s12951-021-01138-2. 34930304 PMC8686281

[157] Wan T, Zhong J, Pan Q, Zhou T, Ping Y, Liu X. Exosome-mediated delivery of Cas9 ribonucleoprotein complexes for tissue-specific gene therapy of liver diseases. Sci Adv. 2022;88:eabp9435. 10.1126/sciadv.abp9435. PMC9473578 36103526

[158] Li H , Zhao S, Jiang M, Zhu T, Liu J, Feng G. et al. Biomodified extracellular vesicles remodel the intestinal microenvironment to overcome radiation enteritis. *ACS Nano* 2023;17:14079–98. 10.1021/acsnano.3c04578. 37399352

[159] Yang J , Liu XX, Fan H, Tang Q, Shou ZX, Zuo DM. et al. Extracellular vesicles derived from bone marrow mesenchymal stem cells protect against experimental colitis via attenuating colon inflammation, oxidative stress and apoptosis. *PLoS One* 2015;10:e0140551. 26469068 10.1371/journal.pone.0140551 PMC4607447

[160] Liu L , Guo S, Shi W, Liu Q, Huo F, Wu Y. et al. Bone marrow mesenchymal stem cell-derived small extracellular vesicles promote periodontal regeneration. *Tissue Eng Part A* 2021;27:962–76. 10.1089/ten.tea.2020.0141. 32962564

[161] Shen Z , Kuang S, Zhang Y, Yang M, Qin W, Shi X. et al. Chitosan hydrogel incorporated with dental pulp stem cell-derived exosomes alleviates periodontitis in mice via a macrophage-dependent mechanism. *Bioact Mater.* 2020;5:1113–26. 10.1016/j.bioactmat.2020.07.002. 32743122 PMC7371600

[162] Lee AE , Choi JG, Shi SH, He P, Zhang QZ, le AD DPSC-derived extracellular vesicles promote rat jawbone regeneration. *J Dent Res* 2023;102:313–21. 10.1177/00220345221133716. 36348514

[163] Xie Y , Guan Q, Guo J, Chen Y, Yin Y, Han X Hydrogels for exosome delivery in biomedical applications. *Gels* 2022;8. 10.3390/gels8060328 PMC9223116 35735672

[164] Nagelkerke A , Ojansivu M, van der Koog L, Whittaker TE, Cunnane EM, Silva AM. et al. Extracellular vesicles for tissue repair and regeneration: evidence, challenges and opportunities. *Adv Drug Deliv Rev* 2021;175:113775. 10.1016/j.addr.2021.04.013. 33872693

[165] Kordelas L , Rebmann V, Ludwig AK, Radtke S, Ruesing J, Doeppner TR. et al. MSC-derived exosomes: a novel tool to treat therapy-refractory graft-versus-host disease. *Leukemia* 2014;28:970–3. 10.1038/leu.2014.41. 24445866

[166] Nassar W , El-Ansary M, Sabry D, Mostafa MA, Fayad T, Kotb E. et al. Umbilical cord mesenchymal stem cells derived extracellular vesicles can safely ameliorate the progression of chronic kidney diseases. *Biomater Res* 2016;20:21. 10.1186/s40824-016-0068-0. 27499886 PMC4974791

[167] Liu H , Wang Y, Hou Y, Li Z Fitness of chassis cells and metabolic pathways for l-cysteine overproduction in Escherichia coli. *J Agric Food Chem* 2020;68:14928–37. 10.1021/acs.jafc.0c06134. 33264003

[168] Yang D , Park SY, Park YS, Eun H, Lee SY Metabolic engineering of Escherichia coli for natural product biosynthesis. *Trends Biotechnol* 2020;38:745–65. 10.1016/j.tibtech.2019.11.007. 31924345

[169] Yan X , Liu XY, Zhang D, Zhang YD, Li ZH, Liu X. et al. Construction of a sustainable 3-hydroxybutyrate-producing probiotic Escherichia coli for treatment of colitis. *Cell Mol Immunol* 2021;18:2344–57. 10.1038/s41423-021-00760-2. 34480146 PMC8484604

[170] Gujrati V , Prakash J, Malekzadeh-Najafabadi J, Stiel A, Klemm U, Mettenleiter G. et al. Bioengineered bacterial vesicles as biological nano-heaters for optoacoustic imaging. *Nat Commun* 2019;10:1114. 10.1038/s41467-019-09034-y. 30846699 PMC6405847

[171] Kim OY , Park HT, Dinh NTH, Choi SJ, Lee J, Kim JH. et al. Bacterial outer membrane vesicles suppress tumor by interferon-γ-mediated antitumor response. *Nat Commun* 2017;8:626. 10.1038/s41467-017-00729-8. 28931823 PMC5606984

[172] Gujrati V , Kim S, Kim SH, Min JJ, Choy HE, Kim SC. et al. Bioengineered bacterial outer membrane vesicles as cell-specific drug-delivery vehicles for cancer therapy. *ACS Nano* 2014;8:1525–37. 10.1021/nn405724x. 24410085

